# Oceanographic connectivity and environmental correlates of genetic structuring in Atlantic herring in the Baltic Sea

**DOI:** 10.1111/eva.12042

**Published:** 2013-02-04

**Authors:** Amber GF Teacher, Carl André, Per R Jonsson, Juha Merilä

**Affiliations:** 1 Department of Biosciences, University of HelsinkiHelsinki, Finland; 2 Centre for Ecology and Conservation, University of ExeterCornwall, UK; 3 Department of Marine Ecology, University of GothenburgTjärnö, Sweden; 4 Ecological Genetics Research Unit, Department of Biosciences, University of HelsinkiHelsinki, Finland

**Keywords:** Baltic Sea, *Clupeaharengus*, fisheries, genetics, herring, microsatellite, selection, transcriptome

## Abstract

Marine fish often show little genetic structuring in neutral marker genes, and Atlantic herring (*Clupea harengus*) in the Baltic Sea are no exception; historically, very low levels of population differentiation (*F*_ST_ ≍ 0.002) have been found, despite a high degree of interpopulation environmental heterogeneity in salinity and temperature. Recent exome sequencing and SNP studies have however shown that many loci are under selection in this system. Here, we combined population genetic analyses of a large number of transcriptome-derived microsatellite markers with oceanographic modelling to investigate genetic differentiation and connectivity in Atlantic herring at a relatively fine scale within the Baltic Sea. We found evidence for weak but robust and significant genetic structuring (*F*_ST_ = 0.008) explainable by oceanographic connectivity. Genetic differentiation was also associated with site differences in temperature and salinity, with the result driven by the locus Her14 which appears to be under directional selection (*F*_ST_ = 0.08). The results show that Baltic herring are genetically structured within the Baltic Sea, and highlight the role of oceanography and environmental factors in explaining this structuring. The results also have implications for the management of herring fisheries, the most economically important fishery in the Baltic Sea, suggesting that the current fisheries management units may be in need of revision.

## Introduction

Intraspecific biodiversity forms the basis for evolution and is important for the long-term viability of populations and thereby also for sustainable harvesting of species of commercial importance (Frankham et al. 2002; Avise 2008; Schindler et al. 2010). Hence, a better understanding of the factors shaping intraspecific biodiversity is one of the key goals in evolutionary and conservation biology (Allendorf and Luikart 2007).

In the marine environment, gene flow by drifting larvae and migrating adults may be extensive, and accordingly, local adaptation in marine fish has traditionally been viewed as rare, with weak population structuring (Waples 1998). However, recent studies show that marine fish may be spatially structured into genetically distinct populations on both wide (e.g. Bradbury et al. 2010) and remarkably fine geographic scales (e.g. Knutsen et al. 2011). Concurrent variation in ecologically important traits (e.g. spawning time, migration patterns etc.) among populations may also indicate adaptive differentiation, possibly affecting resilience to environmental change and exploitation (Hauser and Carvalho 2008). Further, genomic approaches have identified functional genes subjected to natural selection (e.g. Schulte et al. 2000; Andersen et al. 2009; Corander et al. 2013; Lamichhaney et al. 2012), although the molecular mechanism underlying adaptations and the effects on fitness are as yet typically unknown (cf. Barrett and Hoekstra 2011).

Considering the ongoing depletion of most fish populations (Thurstan et al. 2010; Food and Agriculture Organization of the United Nations 2011), spatial scales of population structuring and adaptation in the marine environment need to be clarified (Reiss et al. 2009). Reduced levels of gene flow may result from natural selection acting on long-distance dispersers (Nosil et al. 2005), oceanographic features constraining larval dispersal (Cowen et al. 2006), the existence of cryptic hybrid or tension zones (e.g. Bierne et al. 2011) as well as from behavioural mechanisms (e.g. natal homing) at different life stages (Jones et al. 1999; Svedäng et al. 2007).

The Baltic Sea in the eastern Atlantic Ocean offers a test bed where several environmental factors show steep geographic gradients, e.g. in salinity, temperature and community composition (Ojaveer et al. 2010). There is a temperature gradient from North to South, and a salinity gradient from North to South (with low salinity in the Bothnian Bay) and from East to West (with low salinity in the Gulf of Finland). The salinity gradient ranges from approximately 1 to 30 parts per thousand from the innermost brackish regions of the Baltic Sea, to the entrance to the Baltic Sea which is a fully marine environment (http://www.itameriportaali.fi/en/itamerinyt/en_GB/itamerinyt/).

Considering these environmental clines, local adaptation is to be expected, and several studies have suggested that such adaptations occur in the Baltic region (reviewed in Johannesson and André 2006; Johannesson et al. 2011). For example, Larsen et al. (2007) found that the European flounder (*Platichthys flesus*) had a number of genes that were expressed at significantly different levels in North Sea fish compared to Baltic Sea fish in a reciprocal transplantation experiment mimicking natural local salinities. Likewise, Atlantic cod (*Gadus morhua*) seem to be adapted to local salinity (Larsen et al. 2012) and temperature (Star et al. 2011) conditions. At the same time both flounder and cod show low levels of neutral genetic divergence among populations.

As well as genetic differentiation caused by local adaptation to environmental heterogeneity, physical barriers to gene exchange can also result in population structuring. Oceanographic currents may act as physical barriers to gene exchange in the marine environment. Biophysical models incorporating oceanographic circulation have been applied successfully to landscape/seascape analyses of dispersal barriers and population structure for marine invertebrates (e.g. Galindo et al. 2006; Kool et al. 2010; White et al. 2010). However, only a few studies have employed biophysical models to test whether genetic differentiation correlates to present-day dispersal probabilities in marine fish (but see Christie et al. 2010; Selkoe et al. 2010; Schunter et al. 2011). In the Baltic Sea, biophysical models of dispersal have been used to explore patterns of growth, mortality and recruitment of herring cod and sprat (e.g. Bartsch et al. 1989; Hinrichsen et al. 2005) but no attempts have been made to test for a link between oceanographic circulation and genetic divergence in Baltic Sea fish populations.

The Atlantic herring (*Clupea harengus*) is found throughout the Baltic Sea, allowing for investigation into interactions between the environment and the genome. Most extant herring populations in the Baltic are spring spawning. They produce pelagic larvae that are potentially free to drift with the sea currents (Johannessen and Moksness 1991). Despite this, tagging experiments show that there is a large degree of homing to spawning sites (Aro 1989). Herring also migrate fairly long distances to feed at other times of the year. For instance, herring that spawn near Rügen in Germany migrate to the Kattegat and Skagerrak to feed (Biester 1979; Aro 1989). Migration routes for feeding appear to vary depending on demography, as in years with stock collapses, adult herring have been shown not to leave the coast at all to feed after spawning (Dragesund et al. 1997). Despite these migrations, spawning areas remain consistent (Iles and Sinclair 1982). It seems probable that the homing to spawning sites may represent some form of local adaptation to the environment, and that there should be detectable population structure at spawning time (Ruzzante et al. 2006; Gaggiotti et al. 2009). In spite of the strong environmental variation in the Baltic region, and the spawning site fidelity, it has until recently proved difficult to detect consistent or strong population structure in herring in the Baltic Sea, possibly due to the young age of the Baltic (<9000 kyr) and because herring populations are large, and hence genetic drift is expected to be weak (cf. Cano et al. 2008).

Previous studies using microsatellites, allozymes, and SNPs show low but concordant and temporally stable divergence between Baltic and North Sea herring populations (Bekkevold et al. 2005; Larsson et al. 2007, 2010; Gaggiotti et al. 2009; Limborg et al. 2012). Along the Skagerrak-Kattegat-Baltic salinity gradient there is a pattern of isolation by distance, and a significant correlation between genetic distance and salinity also when geographical distance is corrected for (Bekkevold et al. 2005). Within the Baltic, Jørgensen et al. (2005a) found a correlation between genetic differentiation and both salinity and sea surface temperature, a result driven by samples from Rügen in the southern Baltic. It later became clear that these patterns were strongly influenced by an outlier microsatellite locus, Cpa112, which showed particularly high levels of differentiation in Baltic herring, with a global *F*_ST_ of 0.033, compared to *F*_ST_ values ranging from 0.000–0.007 for the other eight commonly used microsatellite markers (Larsson et al. 2007, 2010; Gaggiotti et al. 2009). These results indicate that this marker may be located near to a selected gene (or regulatory element) and, critically, Cpa112 shows a gradient in allele frequencies from a marine to a brackish environment (André et al. 2011).

Three recent studies on herring using exome sequencing (Lamichhaney et al. 2012) and a panel of SNP markers (Corander et al. 2013; Limborg et al. 2012) have demonstrated a large number of SNPs showing levels of population differentiation (as measured by *F*_ST_) that are consistent with strong positive selection. Divergence between Baltic and North Sea herring populations is pronounced, and samples from the Kattegat cluster separately from Baltic Sea and Atlantic samples, but more closely to Baltic Sea samples (Lamichhaney et al. 2012).

Altogether these previous studies indicate that there is a zone around the Danish Straits where there may be limited gene flow (Gaggiotti et al. 2009), indicative of a hybrid or tension zone in this region between Atlantic and Baltic Sea herring (e.g. Jørgensen et al. 2005a; Limborg et al. 2012). However, within-Baltic structuring remains unclear as the recent exome and SNP studies use few sampling sites within the Baltic. Older studies indicate that Rügen may represent a distinct population although differentiation among temporal samples has been demonstrated both within and between years (Jørgensen et al. 2005a,b; Bekkevold et al. 2007). In addition, the results of Jørgensen et al. (2005a) suggest a possible barrier to gene flow between the Northern and Southern Baltic, though the support for this is relatively weak. Despite these hints at population differentiation, it is clear that we are only beginning to understand population structuring and adaptive divergence in the Baltic herring.

In this study, we took advantage of a large number of newly developed gene-associated microsatellite markers (Teacher et al. 2011) to perform a thorough study of population differentiation in the Atlantic herring in the Baltic region. We further used a biophysical model to predict relative connectivities within the Baltic seascape and explored associations between genetic population structure and dispersal barriers, and how these align with the existing management units. The sampling strategy was designed to cover the Danish Straits region on both sides, including the Skagerrak, Kattegat and the Baltic, and including the potentially critical Rügen area. Specifically, we address the following questions: (i) Is there evidence for population differentiation in the Baltic Sea, (ii) Is there an indication of outlier loci that may be under selection, (iii) Do gene-associated microsatellites show different patterns of population differentiation than random microsatellite markers, (iv) How well does population differentiation correlate with seascape connectivity and geographical distance, and (v) Is there any evidence for correlations between genetic differentiation or genetic diversity with environmental variables, including salinity, temperature, and fishing pressure. Finally, we discuss our findings in the context of fisheries management.

## Methods

### Sampling

**Figure 1:**
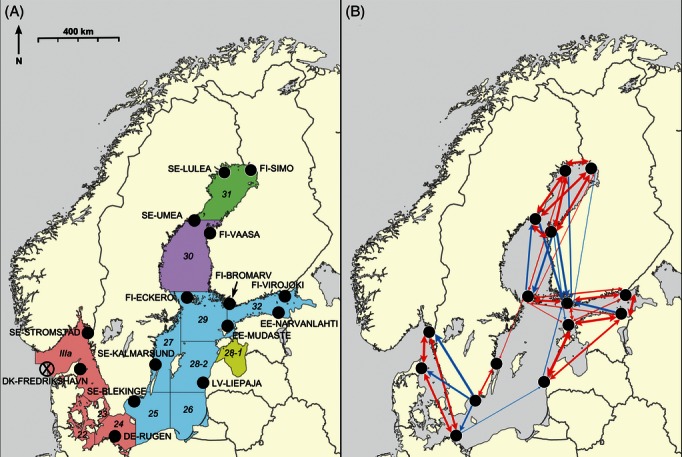
Map showing (A) the locations of the 15 sampling sites and the existing five herring management units in the Baltic, and (B) connectivities among sites based on oceanographic modelling. The direction of North and an approximate scale are also shown. The numbers and the divisions on the sampling site map indicate the ICES fisheries subdivisions, while the colours in the background denote the different management units. The five management units are: Bothnian Bay (ICES fisheries subdivision SD31), Bothnian Sea (SD30), Baltic Sea (SD25–29 & 32 excluding the Gulf of Riga), Gulf of Riga (SD28.1), and Western Baltic together with spring-spawning herring in Kattegat and Skagerrak (SD22–24 and IIIa). The point used for the ‘entrance to the Baltic’ is shown with a cross in a circle (coordinates 57º06′12.85″N, 08º00′59.28″E). On the connectivity map, the line thickness indicates the relative strength of connectivities on a log-scale. Bi-directional edges (links) are coloured red and indicate that connectivities in both directions were above the threshold, while mono-directional blue edges indicate that connectivities were highly asymmetric between locations with one direction below the threshold.

Herring were fished during spring spawning in 2009 or 2010 at 15 sites spread throughout the Baltic Sea and the opening to the Baltic from the North Sea (Fig. 1A, Table S1). The samples used in this study were collected in accordance with the national legislation of the countries concerned. In total, 47 fish from each site were used for genotyping (*n* = 705). Total DNA was extracted from a small segment of fin clip for each individual using a silica-based method (Ivanova et al. 2006).

### Genotyping

In total, 68 microsatellite loci were genotyped, consisting of 51 transcriptome-derived loci (all loci that were listed as polymorphic and reliable for scoring in Teacher et al. 2011), six genome-derived loci that were originally designed for *Clupea harengus* (CHA1005, CHA1017, CHA1020, CHA1027, CHA1059, CHA1202; McPherson et al. 2001), and 11 genome-derived loci for *Clupea pallasii* (CPA101, CPA102, CPA103, CPA104, CPA105, CPA107, CPA108, CPA111, CPA112, CPA113, CPA114; Olsen et al. 2002). Multiplexes of two to five loci were used (Table S2). The PCRs were performed in 8 μL, using 0.8 μL primers (FAM/TET primers at 2 μm and HEX primers at 3 μm), 4 μL 2xPhusion® Flash High-Fidelity PCR Master Mix (Finnzymes), 2.2 μL water, and 1 μL of DNA (~2 ng of DNA). The PCR program was: 98°C for 1 min, followed by 34 cycles of 98°C for 1 s, 58°C for 12 s, 72°C for 20 s, and a final extension at 72°C for 1 min. The PCR products were diluted 1:80, and run on a MegaBace 1000 capillary sequencer. Genotyping was performed using Fragment Profiler 1.2 software (GE Healthcare, Life Sciences, Helsinki, Finland), and automated allele binning was checked manually.

### Data quality checks

Eight samples, each from a different site, were put through PCR and genotyping twice with all loci as controls for error checking. In addition, all samples from the sites FI-VAASA, EE-NARVANLAHTI and DE-RUGEN were run twice through PCR and genotyping to check the results. No genotyping error was detected. The Microsatellite Toolkit (Park 2001) was used to summarise the alleles at each site for each locus. This data was checked manually to identify cases of repeat units that did not match with the repeat motif length, such as one base-pair differences. The remaining loci were run through Microchecker (Van Oosterhout et al. 2004) to check for the presence of null alleles, large allele dropout, and scoring errors caused by stutter patterns, and for linkage disequilibrium (LD) using in Genepop on the Web (Raymond and Rousset 1995), testing all pairs of loci in each site. Six loci (Her53, Her54, Her61, Her100, Her111, CPA102) were rejected during manual checking of Microsatellite Toolkit outputs on the basis of inconsistent and unreliable scoring, leaving 62 loci in the dataset. Two loci (Her28 and CHA1005) showed null alleles in all sites using Microchecker, and these were all significant at 5% after sequential Bonferroni correction. These two loci were removed leaving 60 loci. No locus pairs were shown to be consistently in significant LD across the majority of sites, and so no further loci were removed, leaving a final dataset of 60 loci (45 transcriptome-derived markers, and 15 genome-derived markers). Individuals that were successfully genotyped at fewer than 40 loci out of the total 60 loci were then removed (11 individuals), leaving a final dataset of 694 individuals.

### Outlier tests

Two selection test methods were used: Fdist (Beaumont and Nichols 1996) implemented in Lositan (Antao et al. 2008), and BayeScan (Foll and Gaggiotti 2008). BayeScan has the advantage of allowing estimation of population specific *F*_ST_ values, thus allowing for different demographic histories and drift between populations. Lositan was run using 100 000 simulations, a ‘neutral’ mean *F*_ST_ (potentially non-neutral loci are removed before calculating the initial mean *F*_ST_), confidence intervals of 95%, a false discovery rate (FDR) of 0.05 (implemented within Lositan), with both the Infinite Alleles and the Stepwise mutation models. BayeScan was run with burn in = 50 000, thinning = 50, sample size = 1000, number of iterations = 300 000, number of pilot runs = 20, length of pilot runs = 5000, and an FDR of 0.05 (implemented within BayeScan). Outlier loci were considered to be those that were identified by both methods as being significant outliers. Based on the results of the outlier testing, three datasets were created consisting of: (i) all 60 loci, (ii) 59 loci, excluding the most severe outlier (Her14), and (iii) the most severe outlier, Her14, alone.

BLAST searches were performed for loci Her14 and CPA107 using the NCBI website (http://blast.ncbi.nlm.nih.gov/Blast.cgi). We used blastn searches against the nucleotide collection (nr/nt), reference RNA sequence (refseqrna), and the three-spined stickleback (*Gasterosteus aculaetus*) genome, and blastx searches against non-redundant protein sequences (nr) and reference proteins (refseq_protein).

### Genetic diversity and structure

Allelic richness (*A*_R_, corrected for sample size) and expected heterozygosity (*H*_E_) were calculated for the three different datasets and for each sampling site using FSTAT version 2.9.3.2 (Goudet 1995). A one-way anova was performed using the add-in ‘Analysis ToolPak’ in Excel, to test for differences in *A*_R_ and *H*_E_ between sites. Departures from HWE (heterozygote deficiency) using *F*_IS_ values were tested for each locus in each site using GenePop on the Web (Raymond and Rousset 1995). The average *F*_IS_ with 95% confidence intervals was calculated for each site using Genetix version 4.05 (Belkhir et al. 1996).

Matrices of pairwise *F*_ST_ values based on Weir and Cockerham's (1984) theta estimator of *F*_ST_ were calculated for the three datasets and tested using the exact G-test in Genepop 4.1.0 (Raymond and Rousset 1995). Overall *F*_ST_ was calculated for each locus, and overall *F*_ST_ for all loci over all sites were calculated using GenePop version 4.1.0 (Raymond and Rousset 1995). To visualise the pairwise estimates of *F*_ST_, multidimensional scaling (MDS) plots were generated using the cmdscale function in R (R Development Core Team 2008) for the different datasets.

Structure version 2.3.1 (Pritchard et al. 2000) was used to assign individuals to clusters using no prior information on which sites the individuals belonged to. Structure was run using 800 000 iterations, with a burn in of 400 000, for 10 independent runs for K = 2–10, using correlated allele frequencies, and for both an admixture model and a no-admixture model. These settings were used for the three different datasets. A no-admixture model was thought to be more appropriate due to the regime of sampling only spring spawning herring, where little mixing is thought to occur between spawning regions (Iles and Sinclair 1982; Aro 1989). These analyses were run on the University of Oslo Bioportal cluster (https://www.bioportal.uio.no/). The results files were then used in Structure Harvester (Earl and vonHoldt 2011) in order to estimate the uppermost optimal number of clusters in the datasets using Evanno's delta K method (Evanno et al. 2005). The Structure Harvester outputs for the optimal value of K were then run through CLUMPP (Jakobsson and Rosenberg 2007) using the ‘greedy’ algorithm in order to generate consensus data for the 10 independent runs. Finally, DISTRUCT was used to visualise the data (Rosenberg 2004). As one strong cluster was identified (DE-RUGEN, SE-STROMSTAD, DK-FREDRIKSHAVN, and LV-LIEPAJA), the analyses were repeated excluding this cluster in order to detect any hierarchical structure.

In order to check for the impact of outlier loci on the results, shorter Structure runs (iterations = 40 000, burnin = 40 000) were performed with and without the detected cluster for K = 2–10 for the following datasets: (i) all loci detected as outliers under putative positive selection by either Lositan or BayeScan, (ii) all loci detected as outliers under putative balancing selection, (iii) all loci detected outliers under either positive or putative balancing selection, (iv) all loci except those detected as outliers under putative positive selection, (v) all loci detected as outliers under putative balancing selection, and (vi) all loci except those detected as outliers.

Markers derived from the transcriptome, and thus gene-associated and potentially affected by directional or purifying selection, may show a different level of differentiation compared to loci derived from genomic libraries, and putatively selectively neutral. In order to test this we performed Welch Two Sample *t* tests (unpaired, not assuming equal variance, two-tailed) to test for differences between the mean locus specific *F*_ST_ values, expected heterozygosity, and allelic richness of each marker type.

### Isolation by oceanographic connectivity and geographical distance

Oceanographic connectivity offers a mechanistically derived measure of the potential exchange between populations which may explain patterns of genetic differentiation (White et al. 2010). We modelled relative oceanographic connectivity between the 15 spawning locations using a biophysical model based on ocean circulation and drift trajectories in surface waters. The three-dimensional Rossby Centre Ocean model (RCO) for the Baltic Sea (described in Meier et al. 2003) produced velocity fields in hindcast mode for 25 years (1981–2005) with a horizontal resolution of 3.7 km (2 nm), a vertical resolution of 3–12 m, and a temporal resolution of 6 h. The RCO model is forced by hourly sea level data and climatological mean temperature and salinity profiles from empirical data. Atmospheric forcing is based on the ERA40 data. Precipitation is added every 12 h together with monthly river runoff data. The model has a pseudo-free surface, and is coupled with a multi-layer dynamic-thermodynamic ice model of Hibler type (Hibler 1979). Subgrid turbulent mixing is parameterized using a κ-ε scheme (Meier 2001). The RCO model reproduces velocities, sea surface temperatures, salinity and temperature profiles and ice cover in a satisfactory way (Meier 2001).

Herring connectivity is a function of both passive dispersal of larvae and migratory behaviour in adult fish. The biophysical model we employ here only considers seascape connectivity caused by ocean circulation in surface waters, and the dispersal of herring larvae was simulated as particle trajectories lasting for 3 weeks. Herring larvae hatch and become pelagic at a size of ca. 7 mm and grow to ca. 15 mm in 3 weeks (Arrhenius and Hansson 1996). Larval trajectories were calculated with the Lagrangian trajectory model TRACMASS based on Döös (1995). Trajectories were simulated in off-line mode using the velocity fields generated by the RCO model. Dispersal from each of the 5168 grid cells in the Baltic Sea and Kattegat with a mean depth above 12 m was simulated by releasing 140 particles distributed across one grid cell and at depths between 0–6 m. This was repeated at 25 time points (in spring) within each year and repeated for all 25 years resulting in a total of 87 500 trajectories per grid cell and for the whole model domain 452 200 000 trajectories. Dispersal probabilities between all grid cells (depth ≤12 m) were calculated as the proportion of trajectories starting at grid cell *j* and ending in grid cell *i*, and then summarised in a 5168 × 5168 connectivity matrix. We then multiplied the connectivity matrix by itself 30 times to estimate connectivities among spawning locations for 30 dispersal events, which may be viewed as a multiple-generation connectivity and correspond better to gene flow than single dispersal events (e.g. White et al. 2010). We chose a time scale of 30 generations as a trade-off between an optimal evolutionary times scale and computational constraints, considering the size of the connectivity matrix. The projection over 30 dispersal events produced a connectivity matrix where all elements were non-zero. For each of the 15 spawning locations we selected five neighbouring grid cells and calculated all 210 inter-location connectivities. Note that this calculation considers all possible dispersal routes between two sites for a series of 30 dispersal events. The connectivities between spawning locations were visualised as a network graph. Inter-location probabilities of connectivity varied between 10^−59^–10^−5^, and we arbitrarily applied a threshold for inclusion in the network graph at 10^−20^. A pairwise matrix of directional connectivity resulted from this data. For the purposes of performing an analysis equivalent to an isolation by distance analysis, the between site directional connectivities were averaged to produce a matrix of overall non-directional pairwise connectivity.

The geographical distance between each pair of sites was estimated using the most direct marine route using Google Earth (Google 2011). Isolation by distance tests comparing *F*_ST_ and geographical distance were performed using Mantel tests implemented in R (R Development Core Team 2008). These analyses were performed for the three datasets separately. We also repeated the analyses with each dataset excluding DE-RUGEN as this site appeared to be an outlier (see results). The isolation by distance tests were then repeated using pairwise oceanographic connectivity instead of geographical distance. In addition, partial Mantel tests were performed in R to establish the correlation between *F*_ST_ and geographical distance correcting for oceanographic connectivity, and the between *F*_ST_ and oceanographic connectivity correcting for geographical distance. Finally, a Mantel test was performed to check for covariation between geographical distance and oceanographic connectivity.

### Environmental correlates

The mean temperature and salinity at 5–10 m depth were estimated using Helcom data (http://ocean.ices.dk/Helcom/Default.aspx) for locations closest to the sampling sites. Two different averages were used: mean values for the month of April (providing an estimate of how the values differ at a fixed point in time; April was chosen as it was the most common spawning month, Table S1), and mean values for the 2 weeks spanning the estimated spawning time (collection date was used as the estimated spawning time, as sampling was performed during spawning). In addition, an estimate of fisheries pressure based on ICES (2011) data for mean annual number of tonnes of landed Atlantic herring between 2006–2010 was used. A measure of the estimated shortest marine distance from a point where the Baltic meets the North Sea (coordinates 57º06′12.85″N, 08º00′59.28″E, Fig 1A) was also used as an explanatory variable in the analyses of genetic diversity.

#### Genetic diversity and the environment

Genetic diversity may be reduced due to directional selection in extreme environments (such as very low temperatures or salinity), or due to bottleneck effects such as those induced by high fishing pressure (Frankham et al. 2002). We tested for correlations between genetic diversity and environmental variables using general linear models, implemented in R (R Development Core Team 2008). Allelic richness and expected heterozygosity were fitted as response variables in separate models. The explanatory variables were: mean April temperature, mean April salinity, mean spawning temperature, mean spawning salinity, distance from the entrance to the Baltic, and herring fishery pressure. In addition, interactions between mean temperature and salinity were tested for both the April and spawning time estimates. We used the ‘drop1’ function in R to individually drop each term to remove any effect of the order in which the terms were entered, and checked the residuals for normality. No FDR correction was applied, as the significant results were consistent across different datasets (all sites, and all sites excluding DE-RUGEN).

#### Genetic differentiation and environmental distance

We used Mantel and partial Mantel tests implemented in R to test for correlations between genetic distance (as measured by *F*_ST_) for the three different datasets and environmental distances. Increased environmental distance may result in increased genetic distance as individuals or populations adapt to different environmental factors in different regions (Frankham et al. 2002). The environmental distance matrices were generated using the ‘dist’ function in R for mean spawning time temperature and salinity, and mean April temperature and salinity. Mantel tests were performed between each genetic distance matrix and each environmental distance matrix, and the analyses were then repeated as partial Mantel tests, controlling for geographical distance by sea and then oceanographic connectivity. In order to account for any covariation between temperature and salinity, we performed the following partial Mantel tests for each of the three datasets: Spawning-time temperature controlling for spawning-time salinity and vice-versa, April temperature controlling for April salinity and vice-versa. We repeated all analyses excluding DE-RUGEN. For all analyses, we used the Pearson test with 10 000 permutations.

#### Associations between alleles and environmental variables

In order to test for associations between specific alleles and environmental variables, and to further substantiate both the outlier and environmental correlation tests, MatSAM v.2 (Joost et al. 2008) was used. MatSAM uses multiple univariate logistic regressions to test for associations between allelic frequencies and environmental variables. In total we had 806 alleles which were tested against the following explanatory variables: latitude, distance from the entrance to the Baltic, mean April temperature, mean April salinity, mean spawning time temperature and mean spawning time salinity. The complete dataset and the dataset excluding site DE-RUGEN were both run. Wald statistics were used to assess the associations, with a Bonferroni correction applied within the MatSAM software. The resulting alleles were assessed at three probability levels (*P* = 0.05, 0.0001, and 0.0000001).

### Comparison with previous data

Comparisons were made with previous studies on herring population differentiation in the Baltic (Bekkevold et al. 2005; Jørgensen et al. 2005a) in order to investigate whether the overall *F*_ST_ values were comparable and whether the location DE-RUGEN is consistently distinct, or whether the observed genetic structure may be caused by temporal fluctuations. These comparisons were made using subsets of the nine loci used in the present study that were also used in previous studies (the Jørgensen et al. 2005a study uses one different locus to the Bekkevold et al. 2005 study; loci Cpa107 and Cpa113, respectively). Using these loci, pairwise *F*_ST_ values were identified for three site comparisons where the herring had been sampled in similar locations to in the present study: DE-RUGEN versus SE-STROMSTAD, FI-BROMARV, and FI-SIMO.

## Results

### Outlier tests

The loci detected as outliers by Lositan were the same under the Infinite Alleles and Stepwise mutation models. Those that fell outside the 95% confidence intervals were mostly in the direction of positive selection (High *F*_ST_ relative to *H*_E_): CPA104, CPA107, CPA112, Her14, Her37, Her41, Her63, while locus CPA114 fell in the direction of putative balancing selection (Low *F*_ST_ relative to *H*_E_). Of these, two loci (CPA107 and Her14) were identified as significant outliers after FDR correction (Table S3). BayeScan identified the same two loci as putatively under positive selection, and 20 other loci as potentially being under putative balancing selection (Table S4). It should be noted that while the statistical detection of positive selection is relatively easy and robust with low levels of neutral *F*_ST_ as found here, the detection and interpretation of negative or balancing selection (i.e. lower *F*_ST_ than expected) is difficult and much less robust.

Locus Her14 (*F*_ST_ = 0.075, *α* = 1.521, *P*(*α* ≠ 0) = 1.000) was shown to be considerably more of an outlier than CPA107 (*F*_ST_ = 0.035, *α* = 1.084, *P*(*α* ≠ 0) = 0.963), hence our decision to run specific Her14 analyses. The blastn nr/nt search for locus Her14 against the nucleotide collection only showed hits for the microsatellite repeat region of the sequence, with short flanking regions (total size ≍ 60 base pairs). The blastn search against the three-spined stickleback genome, reference RNA sequences, and the blastx searches also showed no good hits. Locus CPA107 showed no good blastn nr/nt, genome, refseqrna, or blastx hits.

### Genetic diversity and structure

Allelic richness and expected heterozygosity averaged across the 60 loci ranged between 4.804–5.751, and 0.467–0.551, respectively (Table S5). The one-way anova showed that *A*_R_ and *H*_E_ did not differ significantly between sites (*A*_R_: *F*_14,885_ = 0.330, *P* = 0.990; *H*_E_: *F*_14,885_ = 0.353, *P* = 0.986). Estimates of *F*_IS_ and *P*-values for departure from HWE (heterozygote deficiency) showed that no single locus had consistent departure from HWE in all populations (Table S6). Most populations (9 out of 15) showed a homozygote excess, with no apparent geographic pattern (Figure S1).

Overall *F*_ST_ was 0.008 (*P* < 0.001), and ranged from zero to 0.075 for individual loci, with locus Her14 having the highest (*F*_ST_ = 0.075) and locus Her37 the next highest estimate (*F*_ST_ = 0.042; Table S7). Pairwise *F*_ST_ values (Table S8) using all 60 loci showed significant genetic structure between the majority of the sites (80/105 site pairs had significant *F*_ST_ values at the 0.05 FDR), and this result remained the same on removal of locus Her14. For locus Her14 alone, 40/105 site pairs showed a significant *F*_ST_, and the great majority of these were between the sites SE-STROMSTAD, DK-FREDRIKSHAVN, DE-RUGEN, LV-LIEPAJA, and all other sites (38/40 of the significant results).

**Figure 2:**
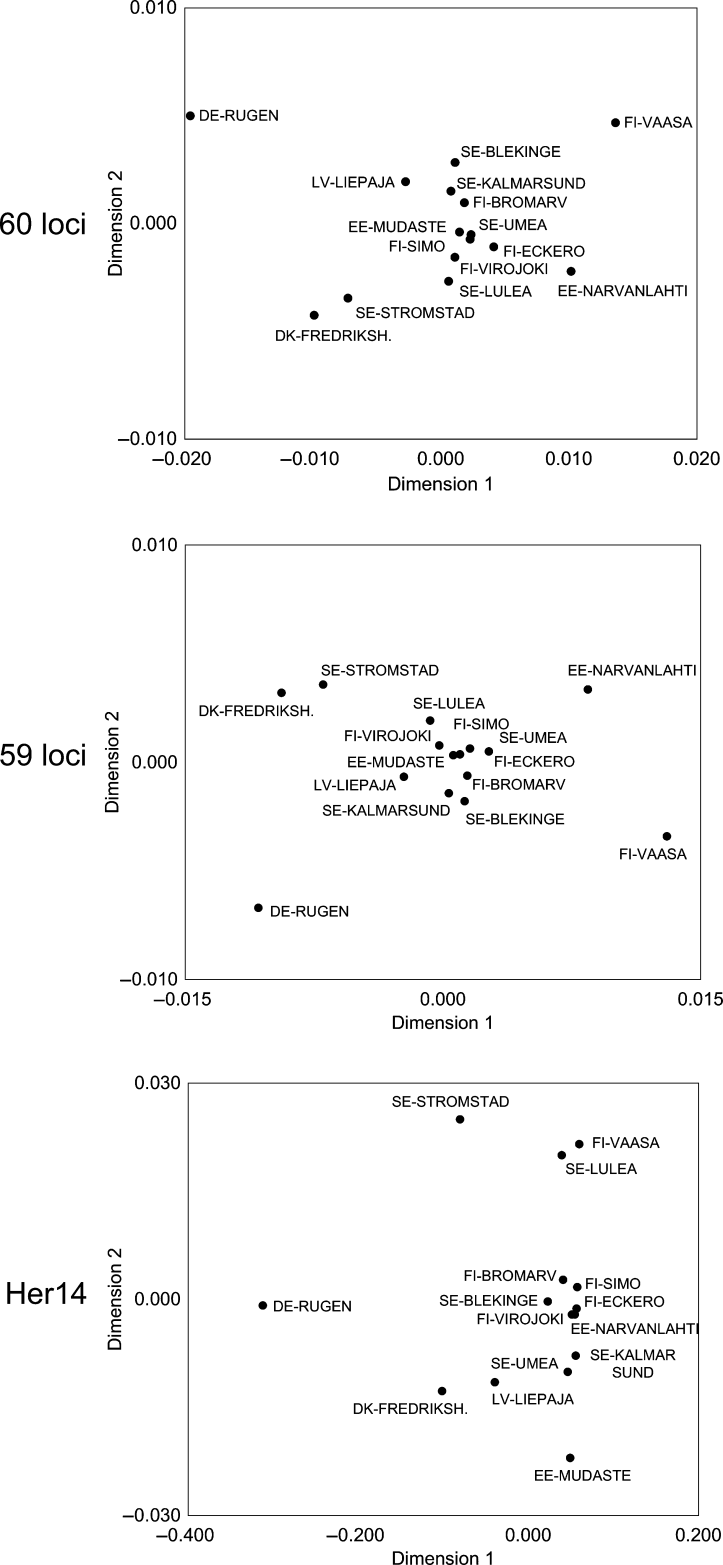
Multidimensional scaling (MDS) plots of herring samples based on pairwise *F*_ST_. Plots show all 60 loci (top), 59 loci (excluding Her14, middle), and the outlier locus Her14 (bottom).

The multidimensional scaling (MDS) plot for all 60 loci (Fig. 2) show that DE-RUGEN is very distinct from the other samples. SE-STROMSTAD and DK-FREDRIKSHAVN cluster together, and EE-NARVANLAHTI and FI-VAASA are outliers in the other direction along dimension 1. The remainder of the samples clustered fairly closely together. The analysis just using the Her14 locus showed a slightly more scattered pattern while still placing DE-RUGEN as an outlier. DK-FREDRIKSHAVN and SE-STROMSTAD were separated, and SE-LULEA, FI-VAASA and EE-MUDASTE were outliers along dimension 2 (Fig. 2). The results from the dataset consisting of 59 loci (excluding Her14) were very similar to those for the 60 loci dataset.

**Figure 3:**
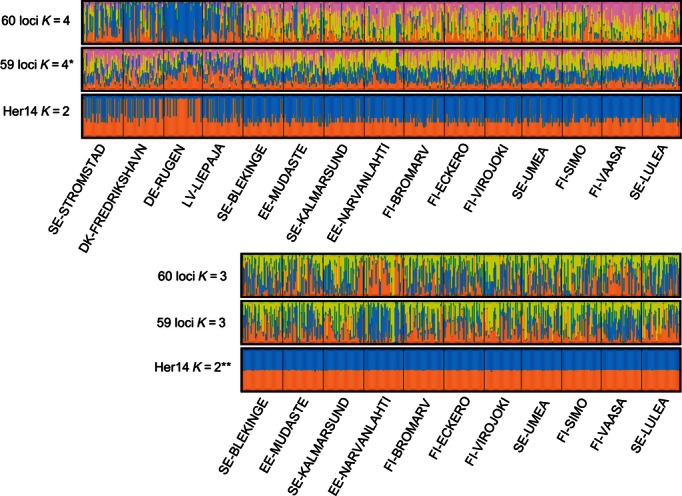
Bayesian clustering results from Structure. Bar plots are shown for all 60 loci, 59 loci (excluding Her14), and the outlier locus Her14. Plots are shown for the most likely number of clusters for each dataset (delta-K analyses), for all sites (top three plots) and excluding the DE-RUGEN, DK-FREDRIKSHAVEN, SE-STROMSTAD and LV-LIEPAJA cluster (bottom three plots). Within each plot, each vertical bar represents an individual, clusters are indicated by colour, and the *y*-axis of each plot shows the proportion of the genotype for each individual belonging to each cluster. The plot marked with a star (*) indicates that this analysis failed to converge for the delta-K analysis and so the value of K was instead chosen based on log-likelihood values. The plot marked with two stars (**) indicates that although the mostly likely value of K is 1, it is not possible to assess this possibility using Structure, and so the plot for K = 2 is shown. Probability values below 0.05 are shown in bold.

The Bayesian clustering analyses using Structure showed that when using the dataset of all 60 loci with no admixture, the sites DE-RUGEN, SE-STROMSTAD, DK-FREDRIKSHAVN, and LV-LIEPAJA stand out as a distinct cluster (Fig. 3). DE-RUGEN is the most distinct site, and SE-STROMSTAD, DK-FREDRIKSHAVN, and LV-LIEPAJA have a high proportion of individuals of this type. It is worth noting that DE-RUGEN (located within the Baltic Sea) is more divergent from the other Baltic Sea populations than are SE-STROMSTAD and DK-FREDRIKSHAVN (located outside of the Baltic Sea). In addition, when the DE-RUGEN cluster was excluded from the analysis, the EE-NARVANLAHTI and FI-VAASA sites appear to form a weak second cluster as for the MDS plots (for the 60 locus and 59 locus datasets, not with Her14 alone). Locus Her14 is clearly important for the differentiation of the DE-RUGEN, SE-STROMSTAD, DK-FREDRIKSHAVN, LV-LIEPAJA cluster, although the inclusion of LV-LIEPAJA is less clear when only looking at this single locus. When locus Her14 was removed resulting in a dataset of 59 loci, the results were very similar to those of 60 loci (Fig. 3), indicating that the effect is not solely driven by Her14. The number of clusters suggested by Evanno's Delta K (Evanno et al. 2005) was K = 4 for all 60 loci, undetermined for 59 locus dataset, and K = 2 for locus Her14 (Figure S2). The complete analysis for the 59 locus dataset was repeated three times with no change to the result; the Structure analysis converged and the log likelihood values supported K = 4 (as for the 60 locus dataset), however the Delta K analysis resulted in multiple peaks (Figure S2). When allowing for full admixture, the Her14 dataset shows the same clusters as for the ‘no admixture’ analyses, however all other datasets show no structure (data not shown). Structure results with the outlier loci removed (2 outlier loci under putative positive selection, 20 outlier loci under putative balancing selection, or both) showed much the same results as the 60 and 59 loci datasets (Figure S3). The loci thought to be under putative balancing selection alone show no obvious structure, the loci thought to be under positive selection alone show similar results as the Her14 dataset, and all outlier loci together again show similar results to the Her14 dataset, indicating that none of the other outlier loci are having a substantial effect on the clustering results. It should once again be noted that inference relating to loci under putative balancing selection is unreliable due to the low power to detect such loci; however, this analysis serves to rule out any substantial influence of these loci to the population structure results.

When only the 38 putatively neutral loci are run (i.e. all outliers removed), both the DE-RUGEN, SE-STROMSTAD, DK-FREDRIKSHAVN, LV-LIEPAJA cluster, and the EE-NARVANLAHTI, FI-VAASA cluster are still identified (Figure S3). Again, DE-RUGEN remains more divergent than SE-STROMSTAD and DK-FREDRIKSHAVN from the Baltic Sea populations.

Comparisons of the different marker types indicated that there was no difference between the mean locus-specific *F*_ST_ values for the transcriptome versus the genome-derived microsatellite markers (*t*_34.664_ = 0.131, *P* = 0.896), however expected heterozygosity and allelic richness were both significantly lower in the transcriptome-derived loci (*H*_E_ transcriptome mean = 0.436, *H*_E_ genome mean = 0.764, *t*_28.554_ = −5.403, *P* < 0.001; *A*_R_ transcriptome mean = 4.280, *A*_R_ genome mean = 9.483, *t*_18.170_ = −4.973, *P* < 0.001, Table S7).

### Isolation by oceanographic connectivity and geographical distance

The map illustrating the multiple-generation connectivities among the sites based on oceanographic modelling indicates three main regions of connectivity, one in the North around the Bothnian Bay, one in the East around the Gulf of Finland, and one in the West around the Kattegat/Skagerrak (Fig. 1B).

Significant isolation by distance (IBD) was found using both the dataset with all 60 loci (*r* = 0.317, *P* = 0.040), and 59 loci (*r* = 0.324, *P* = 0.025), but not with Her14 (*r* = 0.258, *P* = 0.095; Table 2 and Figure S4). As DE-RUGEN appeared to be so distinct from the other sites, our analyses were run both including and excluding this site. When the site DE-RUGEN was removed, it became apparent that both this site, and locus Her14 strongly influenced the results. In the tests without DE-RUGEN, stronger IBD was found using all 60 loci (*r* = 0.431, *P* = 0.005), 59 loci (*r* = 0.368, *P* = 0.020), and Her14 (*r* = 0.628, *P* < 0.001).

Oceanographic connectivity seemed better than IBD at explaining the genetic differentiation: with reduced genetic differentiation where there was high oceanographic connectivity (60 loci: *r* = 0.413, *P* = 0.004; 59 loci: *r* = 0.381, *P* = 0.004; Her14: *r* = 0.399, *P* = 0.007). The correlations with oceanographic connectivity remained significant when the DE-RUGEN site was removed (60 loci: *r* = 0.386, *P* = 0.010; 59 loci: *r* = 0.342, *P* = 0.026, Her14 *r* = 0.657, *P* = 0.002; Table 2), indicating that connectivity at DE-RUGEN was important but not driving the result exclusively. However, partial Mantel tests showed that geographic distance and oceanographic connectivity strongly covary (including DE-RUGEN *r* = 0.861, *P* < 0.001, excluding DE-RUGEN *r* = 0.860, *P* < 0.001), and only Her14 including DE-RUGEN remained significantly correlated to oceanographic connectivity when geographic distance was accounted for (*r* = 0.359, *P* = 0.035).

### Environmental correlates

Plots of the environmental data show that the two salinity estimates (mean April salinity, and spawning-time salinity) were very similar (Figure S5). The two temperature estimates are less similar to eachother; mean April temperatures (ca. 0–6°C) tended to decrease towards the North, whereas mean spawning-time temperatures (ca. 2–10°C) were more variable between sites.

#### Genetic diversity and the environment

The general linear models showed that locus Her14 heterozygosity was correlated with mean April temperature (*F*_1,6_ = 14.518, *P* = 0.009), and this correlation became a little stronger if the site DE-RUGEN is removed (*F*_1,6_ = 17.872, *P* = 0.008). There were no other statistically significant (*P* < 0.05) correlations (Table S9).

#### Genetic differentiation and environmental distance

In analyses using all sites, no statistically significant correlations were detected between genetic distance (*F*_ST_) and environmental distances (spawning temperature and salinity, April temperature and salinity). However, when DE-RUGEN was removed, it became clear that both this site, and locus Her14 were again having a substantial effect on the results.

Salinity correlated with genetic distance in all datasets without DE-RUGEN (Table 2). After correcting the spawning-time salinity for spawning-time temperature, all the results without DE-RUGEN remained significant (60 loci *r* = 0.486, *P* = 0.007; 59 loci *r* = 0.448, *P* = 0.020; Her14 *r* = 0.900, *P* = 0.001; Table 2), whereas after correcting the April salinity for April temperature, the results only remained significant in the Her 14 dataset (*r* = 0.140, *P* = 0.006; Table 2). The salinity correlations without DE-RUGEN were no longer statistically significant after controlling for geographical distance, however they were significant after correcting for oceanographic connectivity in the 60 locus dataset (spawning-time salinity *r* = 0.348, *P* = 0.038; April salinity *r* = 0.353, *P* = 0.038) and Her14 (spawning-time salinity *r* = 0.821, *P* = 0.005; April salinity *r* = 0.825, *P* = 0.005; Table 2).

April temperature correlated significantly with genetic distance in all datasets without DE-RUGEN (Table 2). After correcting for April salinity, the 60 loci and Her14 results remained significant (60 loci *r* = 0.326, *P* = 0.019; Her14 *r* = 0.412, *P* = 0.034; Table 2), and similarly after correcting for geographical distance the result remained significant in the 60 loci (*r* = 0.348, *P* = 0.021) and locus Her14 datasets (*r* = 0.447, *P* = 0.004; Table 2). After correcting for oceanographic connectivity, only the 60 loci dataset remained significant (*r* = 0.366, *P* = 0.009; Table 2). Spawning-time temperature did not correlate with genetic distance in any dataset.

Corrections for multiple testing were not applied to the Mantel tests due to the consistency of the results across many related datasets (60 loci, 59 loci, Her 14, with and without DE-RUGEN).

#### Associations between alleles and environmental variables

**Figure 4:**
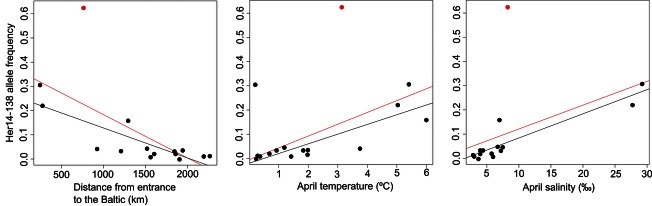
Correlations between allele 138 at locus Her14 and environmental variables. Plots are shown for distance from the entrance to the Baltic, April temperature and April salinity. The point marked in red is site DE-RUGEN, and the trend line in red includes site DE-RUGEN while the trend line in black excludes DE-RUGEN.

The MatSAM analyses revealed that several of the loci identified as outliers by BayeScan or Lositan had allelic frequencies that were associated with environmental variation (Table 1). While nine loci showed associations between allelic frequency and environmental factors at *P* = 0.05, only three of the loci (Her14, CPA112, and Her63) showed such associations at *P* = 0.0001 (after a Bonferroni correction applied within the MatSAM software). It is noteworthy that these analyses show similar results for locus CPA112 associations with salinity as were found by André et al. (2011); the allele 302 in the present study is the same as the allele 306 in the André et al. (2011) study. At the lowest probability value (*P* = 0.0000001), the frequency of allele 138 in locus Her14 was associated with latitude, distance from the entrance to the Baltic, April temperature, and April and Spawning time salinity (Fig. 4). The frequency of this allele increased as temperature and salinity increased, and decreased with decreasing distance from the entrance to the Baltic as well as with decreasing latitude (Fig. 4). The association with latitude is no longer present in the dataset with DE-RUGEN excluded. The frequency of allele 104 in locus Her63 increased with increasing salinity. The opposite pattern was observed for allele 302 in locus CPA112, which decreases with increasing salinity, as also observed by André et al. (2011).

**Table 1:** Results from MatSAM analysis identifying associations between allelic frequencies at marker loci and environmental variables

Locus	Allele	Variable	All sites			DE-RUGEN excluded		
	
			*P* = 0.05	*P* = 0.0001	*P* = 0.0000001	*P* = 0.05	*P* = 0.0001	*P* = 0.0000001
Her14	138	Latitude	SIG	SIG	SIG	SIG	–	–
		Distance from entrance to Baltic	SIG	SIG	SIG	SIG	SIG	SIG
		April temperature	SIG	SIG	SIG	SIG	SIG	SIG
		April salinity	SIG	SIG	SIG	SIG	SIG	SIG
		Spawning temperature	–	–	–	–	–	–
		Spawning salinity	SIG	SIG	SIG	SIG	SIG	SIG
	126	Latitude	SIG	–	–	–	–	–
		Distance from entrance to Baltic	SIG	SIG	–	SIG	–	–
		April temperature	SIG	–	–	SIG	–	–
		April salinity	SIG	–	–	SIG	–	–
		Spawning temperature	–	–	–	–	–	–
		Spawning salinity	SIG	–	–	SIG	–	–
CPA112	302	Distance from entrance to Baltic	SIG	SIG	–	SIG	–	–
		April temperature	SIG	–	–	SIG	–	–
		April salinity	SIG	SIG	–	SIG	SIG	–
		Spawning salinity	SIG	SIG	–	SIG	SIG	–
	333	Distance from entrance to Baltic	SIG	–	–	SIG	–	–
		April temperature	SIG	–	–	–	–	–
		April salinity	SIG	–	–	SIG	–	–
		Spawning salinity	SIG	–	–	SIG	–	–
	Her63	104	Distance from entrance to Baltic	SIG	–	–	SIG	–	–
			April salinity	SIG	SIG	–	SIG	SIG	–
			Spawning salinity	SIG	SIG	–	SIG	SIG	–
	CPA107	194	April salinity	SIG	–	–	SIG	–	–
Spawning salinity			SIG	–	–	SIG	–	–
Her18	178	April salinity	SIG	–	–	SIG	–	–
		Spawning salinity	SIG	–	–	SIG	–	–
Her77	164	Distance from entrance to Baltic	SIG	–	–	–	–	–
Her37	118	April temperature	SIG	–	–	SIG	–	–
Her126	226	Distance from entrance to Baltic	SIG	–	–	–	–	–
		April salinity	–	–	–	SIG	–	–
		Spawning salinity	–	–	–	SIG	–	–
Her43	138	Distance from entrance to Baltic	SIG	–	–	–	–	–

All loci, alleles, and environmental variables with significant results are shown, at three significance levels (*P* = 0.05, 0.0001, 0.0000001; Bonferroni correction applied within the MatSAM software). Results are shown both for the complete dataset and for the dataset with DE-RUGEN excluded.

**Table 2:** Results of Mantel and partial Mantel tests correlating genetic distance with environmental distances

			Geographical distance		Connectivity		Spawning temperature		Spawning salinity		April temperature		April salinity	
					
			*r*	*P*	*r*	*P*	*r*	*P*	*r*	*P*	*r*	*P*	*r*	*P*
60 loci	Mantel	All pops	0.317	**0.040**	0.413	**0.004**	−0.102	0.738	0.272	0.145	0.254	0.087	0.280	0.139
		Excl. DE-RUGEN	0.431	**0.005**	0.386	**0.010**	−0.174	0.885	0.501	**0.003**	0.510	**0.002**	0.504	**0.002**
	Partial - distance	All pops	NA	NA	0.289	0.055	−0.091	0.702	0.080	0.289	0.094	0.235	0.094	0.285
		Excl. DE-RUGEN	NA	NA	0.033	0.412	−0.135	0.806	0.300	0.074	0.348	**0.021**	0.306	0.062
	Partial - connectivity	All pops	−0.082	0.666	NA	NA	−0.086	0.690	0.029	0.369	−0.015	0.421	0.040	0.353
		Excl. DE-RUGEN	0.211	0.126	NA	NA	−0.138	0.818	0.348	**0.038**	0.366	**0.009**	0.353	**0.038**
	Partial - spawning salinity	All pops	NA	NA	NA	NA	−0.066	0.624	NA	NA	NA	NA	NA	NA
		Excl. DE-RUGEN	NA	NA	NA	NA	−0.103	0.749	NA	NA	NA	NA	NA	NA
	Partial - spawning temperature	All pops	NA	NA	NA	NA	NA	NA	0.261	0.149	NA	NA	NA	NA
		Excl. DE-RUGEN	NA	NA	NA	NA	NA	NA	0.486	**0.007**	NA	NA	NA	NA
	Partial - April temperature	All pops	NA	NA	NA	NA	NA	NA	NA	NA	NA	NA	0.178	0.187
		Excl. DE-RUGEN	NA	NA	NA	NA	NA	NA	NA	NA	NA	NA	0.316	0.08
	Partial - April salinity	All pops	NA	NA	NA	NA	NA	NA	NA	NA	0.13	0.166	NA	NA
Excl. DE-RUGEN		NA	NA	NA	NA	NA	NA	NA	NA	0.326	**0.019**	NA	NA
59 loci	Mantel	All pops	0.324	**0.025**	0.381	**0.004**	−0.122	0.788	0.330	0.061	0.288	0.051	0.337	0.063
		Excl. DE-RUGEN	0.368	**0.020**	0.342	**0.026**	−0.140	0.817	0.461	**0.015**	0.428	**0.007**	0.464	**0.013**
	Partial - distance	All pops	NA	NA	0.212	0.113	−0.112	0.763	0.158	0.223	0.134	0.233	0.170	0.212
		Excl. DE-RUGEN	NA	NA	0.381	0.054	−0.102	0.731	0.301	0.716	0.278	0.080	0.307	0.079
	Partial - connectivity	All pops	−0.009	0.516	NA	NA	−0.109	0.755	0.134	0.254	0.063	0.316	0.144	0.258
		Excl. DE-RUGEN	0.154	0.212	NA	NA	−0.104	0.743	0.329	0.056	0.283	0.073	0.334	0.053
	Partial - spawning salinity	All pops	NA	NA	NA	NA	−0.079	0.682	NA	NA	NA	NA	NA	NA
		Excl. DE-RUGEN	NA	NA	NA	NA	−0.07	0.656	NA	NA	NA	NA	NA	NA
	Partial - spawning temperature	All pops	NA	NA	NA	NA	NA	NA	0.318	0.071	NA	NA	NA	NA
		Excl. DE-RUGEN	NA	NA	NA	NA	NA	NA	0.448	**0.02**	NA	NA	NA	NA
	Partial - April temperature	All pops	NA	NA	NA	NA	NA	NA	NA	NA	NA	NA	0.228	0.143
		Excl. DE-RUGEN	NA	NA	NA	NA	NA	NA	NA	NA	NA	NA	0.306	0.089
	Partial - April salinity	All pops	NA	NA	NA	NA	NA	NA	NA	NA	0.138	0.221	NA	NA
		Excl. DE-RUGEN	NA	NA	NA	NA	NA	NA	NA	NA	0.237	0.100	NA	NA
Her14	Mantel	All pops	0.258	0.095	0.399	**0.007**	−0.044	0.530	0.157	0.134	0.168	0.175	0.165	0.132
		Excl. DE-RUGEN	0.628	**0.003**	0.657	**0.002**	−0.148	0.860	0.903	**0.001**	0.637	**0.003**	0.905	**0.001**
	Partial - distance	All pops	NA	NA	0.359	**0.035**	−0.032	0.483	−0.028	0.437	0.027	0.269	−0.015	0.397
		Excl. DE-RUGEN	NA	NA	0.295	0.061	−0.091	0.685	0.837	**0.002**	0.418	**0.036**	0.840	**0.004**
	Partial - connectivity	All pops	−0.182	0.822	NA	NA	−0.023	0.439	−0.117	0.780	−0.124	0.860	−0.108	0.711
		Excl. DE-RUGEN	0.164	0.184	NA	NA	−0.088	0.670	0.821	**0.005**	0.346	0.077	0.825	**0.005**
	Partial - spawning salinity	All pops	NA	NA	NA	NA	−0.021	0.419	NA	NA	NA	NA	NA	NA
		Excl. DE-RUGEN	NA	NA	NA	NA	0.016	0.380	NA	NA	NA	NA	NA	NA
	Partial - spawning temperature	All pops	NA	NA	NA	NA	NA	NA	0.152	0.133	NA	NA	NA	NA
		Excl. DE-RUGEN	NA	NA	NA	NA	NA	NA	0.9	**0.001**	NA	NA	NA	NA
	Partial - April temperature	All pops	NA	NA	NA	NA	NA	NA	NA	NA	NA	NA	0.091	0.14
		Excl. DE-RUGEN	NA	NA	NA	NA	NA	NA	NA	NA	NA	NA	0.864	**0.006**
	Partial - April salinity	All pops	NA	NA	NA	NA	NA	NA	NA	NA	0.097	0.107	NA	NA
		Excl. DE-RUGEN	NA	NA	NA	NA	NA	NA	NA	NA	0.412	**0.034**	NA	NA

The Mantel statistic *r* and associated probability value *P* are shown for Mantel tests correlating population pair-wise *F*_ST_ of three datasets (60 loci, 59 loci, Her14) with the shortest geographical distance by sea (Geographical distance), oceanic connectivity (Connectivity), Spawning-time temperature and salinity, and April temperature and salinity. In addition, results of partial Mantel tests are shown, controlling for geographical distance by sea or oceanic connectivity, as well as controlling temperature results for salinity, and controlling salinity results for temperature, for both spawning-time and April estimates. Probability values below 0.05 are shown in bold

### Comparison with previous data

Temporal comparisons with previous studies with similar geographical coverage using the same nine microsatellite loci showed similar overall *F*_ST_ estimates: 0.006 (2009/10; present study), 0.006 (2002/3; Larsson et al. 2010) and 0.007 (1979/80; Larsson et al. 2010). Specific geographical comparisons between DE-RUGEN and SE-STROMSTAD regions (Bekkevold et al. 2005), as well as inner Baltic regions FI-SIMO and FI-BROMARV (Jørgensen et al. 2005a) corroborate that the Rügen spawning site is genetically distinct (Table S10).

## Discussion

Our results appear to be very robust (genotyping error rate of 0%), and indicate significant genetic structuring in herring within the Baltic Sea. As well as significant pairwise differentiation between the majority of the sites sampled, we also identified several distinct genetic clusters. We found oceanographic connectivity to be a better predictor than geographical distance at explaining the genetic differentiation. A single transcriptome-derived microsatellite (Her14) showed particularly high *F*_ST_ values, indicating possible spatially variable selection in a genomic region close to this locus. Moreover, genetic diversity and differentiation at this microsatellite locus were correlated with local differences in water temperature and salinity.

### Genetic structure

Recent studies have demonstrated clear differentiation between herring in the Atlantic and the Baltic Sea, however these studies have provided limited information on structuring within the Baltic Sea (Lamichhaney et al. 2012; Limborg et al. 2012; see also Corander et al. 2013). Genetic structure has to date been hard to detect in herring in the Baltic Sea, with typical *F*_ST_ values of ≍ 0.008 (Bekkevold et al. 2005; Larsson et al. 2007), though one microsatellite marker (Cpa112) has shown higher levels of differentiation with *F*_ST_ = 0.033 (Larsson et al. 2007, including several North Sea samples; 0.020 in the present study, Table S6). In our study, using a considerably higher (>6 times) number of microsatellite markers, we also found an overall *F*_ST_ of 0.008, with several outlier markers with comparable *F*_ST_ values to Cpa112. However, one marker (Her14), was a substantially stronger outlier with an *F*_ST_ of over double that of Cpa112 (Her14 *F*_ST_ = 0.075). Significant pairwise *F*_ST_ values between the majority of the study sites using all datasets (60 loci, 59 loci and Her 14 datasets) indicated that the genetic differentiation is not driven by a single outlier site. Indeed, clear patterns of isolation by distance confirmed that genetic differentiation is occurring throughout the Baltic region.

While the general pattern was of significant genetic differentiation between the majority of the sites, some distinct clusters were also apparent. The most striking was that of Rügen in the western Baltic (DE-RUGEN) clustering with nearby sites in the Kattegat, Skagerrak and on the Latvian coast (SE-STROMSTAD, DK-FREDRIKSHAVN and LV-LIEPAJA). This cluster was apparent using Bayesian clustering, and also from multidimensional scaling plots of *F*_ST_. While Rügen was the most differentiated site, the other three sites had a large number of individuals that showed similar genotypes to the Rügen individuals. It is perhaps surprising that other nearby sites on the Swedish coast (SE-BLEKINGE, SE-KALMARSUND) do not show such similarity to Rügen. Our results suggest that oceanographic connectivity explains the genetic differentiation better than geographic isolation by distance does, and it can be seen from the biophysical model of ocean connectivity (Fig. 1B) that there is strong bi-directional connectivity between DE-RUGEN and both SE-STROMSTAD and DK-FREDRIKSHAVEN. There is also some uni-directional connectivity from DE-RUGEN to LV-LIEPAJA, wheras there is little or no connectivity from Rügen to the nearby sites SE-BLEKINGE and SE-KALMARSUND. A likely scenario is that passive larval drift occurred from Rügen along the southern Baltic coast bringing alleles originating from Kattegat and Skagerrak, resulting in the observed genetic structure. Limborg et al. (2012) observed a similar pattern for SNP markers with a stronger introgression of North Sea herring alleles along the southern side of the Baltic. Asymmetric gene flow between the northern and southern side of the entrance of the Baltic has been demonstrated also for the free-spawning bivalve *Macoma balthica* and is in accordance with the oceanographic circulation pattern in the Baltic Sea (Elken and Matthäus 2008). Adult herring that spawn in the Rügen area migrate in high numbers to the Skagerrak to feed in the summer, and straying may further contribute to genetic connectivity within this cluster (Biester 1979; Bekkevold et al. 2011). The microsatellite locus that was the strongest outlier (Her14) could be used alone to differentiate the Rügen site and also to some degree the connected sites. When this outlier locus was removed from the dataset, the differentiation of this cluster was reduced slightly, but still held. It may be that there is local adaptation at Rügen which involves either the gene in which this marker is located, or a locus linked to it, or, alternatively, intrinsic genetic incompatability between Rügen herring and inner Baltic herring populations (Bierne et al. 2011; see below). It should be noted, however, that this cluster of four sites also remains distinct when considering only the 38 “neutral” loci (Figure S1), underlining that there is demographic isolation.

Other microsatellite studies on herring have also found Rügen to be an outlier (Bekkevold et al. 2005; Jørgensen et al. 2005a). It is important to note that the Jørgensen et al. (2005a) study found temporal variation at Rügen and the Åland archipelago (sampling site very close to our FI-BROMARV site) samples, but not at other locations. This was attributed to different spring spawning waves occurring within each of these sites, and a later study revealed temporal genetic differentiation at Rügen within the spring spawning season (Jørgensen et al. 2005b). However, Larsson et al. (2010) showed temporally stable patterns of differentiation among Skagerrak, northern Baltic and Bothnian bay herring populations over a 24 year period using the same set of nine microsatellite loci, suggesting a persistent large scale population structure. We compared our results with those from previous studies, limiting our analyses to the same nine microsatellite marker set, and found remarkably similar *F*_*ST*_ estimates between comparable sites from sampling in 2002/2003 (Bekkevold et al. 2005; Jørgensen et al. 2005a) and our sampling in 2009/2010. This indicates that our results are unlikely to be simply caused by temporal fluctuations in allele frequencies (cf. Waples 1998).

It is noteworthy that the genetically distinct DE-RUGEN cluster coincides with the environmental transition zone between the North and Baltic Seas. Previous studies of herring (Gaggiotti et al. 2009; André et al. 2011; Lamichhaney et al. 2012; Limborg et al. 2012), as well as of other species (reviewed by Johannesson and André 2006; see also Luttikhuizen et al. 2012; Bourret et al. 2012), show a distinct genetic break in this area. Furthermore, Lamichhaney et al. (2012) demonstrate that geographically intermediate samples from the Kattegat form a unique cluster, grouping most closely with the Baltic herring. Such breaks can be indicative of primary or secondary hybridization and tension zones (Barton and Hewitt 1985). Bierne et al. (2011) showed that tension zones resulting from genetic incompatibilities between populations with different genetic backgrounds often stabilise at natural environmental barriers making it difficult to determine if outlier loci, with elevated *F*_ST_, result from environmental selection or are due to endogenous forces such as pre- and postzygotic incompatibilities. In low-density genome scans, such as in our case and e.g. the SNP study by Limborg et al. (2012), essentially neutral loci may be in varying linkage with genes having incompatible alleles whereas the specific ‘isolation’ loci may themselves go undetected. Further, barriers to gene flow are often both endogenous and environmentally dependent (Bierne et al. 2011) and to disentangle these factors more information is needed about outlier gene function, the genomic context and historical contingency (e.g. Jones et al. 2012). Presently, we can not fully determine the factors responsible for the outliers in Atlantic herring in the transition zone. However, some of the outliers show similar allele frequency shifts also in other low-salinity areas implying an environmentally induced response (André et al. 2011; Limborg et al. 2012). The recent exome sequencing study by Lamichhaney et al. (2012) shows that many loci that appear to be under positive selection are clustered within certain chromosomal regions, and many of these could be assigned to specific candidate genes that are thought to be under salinity-induced selection (e.g. ATPase proton pump which is involved in osmoregulation).

In addition to the strong cluster discussed above, a second weaker genetic cluster was also identified, grouping sites EE-NARVANLAHTI with FI-VAASA. This cluster is harder to explain as the sites are relatively isolated from each other geographically, with several sampling sites in between (e.g. FI-BROMARV, FI-ECKERO) that do not cluster in the same group. In addition, there is little evidence for oceanographic connectivity occurring exclusively between these sites. It is possible that the similarity could be due to chance, or perhaps due to similar but unknown selective pressures in the two regions (e.g. pollution or disease). Jørgensen et al. (2005a) found indications of a barrier to migration between northern and southern Baltic sites, and the sites that are found in our second cluster both fall within the Jørgensen et al. (2005a) northern Baltic sampling region. However, Jørgensen et al.'s (2005a) northern cluster included samples from the northern part of the Swedish East coast and from the Finnish South coast, while in our study these regions do not group with our second cluster. It is possible that the Northern/Eastern Baltic does in fact represent a distinct area, and perhaps there is a degree of temporal variation that has resulted in the differences between the studies. The biophysical model of ocean connectivity does indicate that the Northern and Eastern Baltic region is somewhat separate from the Southern and Western region (Fig. 1B), lending support to this possibility. Future targeted sampling in this region to fill in the geographical gaps, and sampling over consecutive years may prove useful for understanding this further.

Our comparison of microsatellite marker types indicate that loci derived from the transcriptome had similar *F*_ST_ but significantly lower allelic richness and expected heterozygosity compared to microsatellites derived from genomic libraries. The lower diversity may be explained by purifying selection acting upon the transcribed genes, resulting in relatively more conserved genetic regions (see Bouck and Vision 2007).

### Environmental correlates

Genetic diversity was quite uniform throughout the Baltic and in most tests average *A*_R_ and *H*_E_ did not correlate with environmental factors or fishing pressure. However, when looking at the outlier locus Her14 alone, we did find correlations between heterozygosity and mean April temperature, both when DE-RUGEN was included and excluded from the analysis. In addition, we found a correlation between mean April temperature and population differentiation (*F*_ST_) in all three datasets (when DE-RUGEN was excluded). The *F*_ST_-mean April temperature correlations remained after correcting for geographical distance (60 loci and Her14 datasets only) or oceanographic connectivity (60 loci dataset only), and after correcting for April salinity (60 locus and Her14 datasets only), indicating that the result is likely to be biologically meaningful. No correlations were detected between spawning-time temperature and *F*_ST_ or genetic diversity, and this is likely because the temperature at the spawning time is less reflective of differences between the sites: mean April temperatures reflect differences between the sites at a fixed time, while the spawning dates are not fixed between sites. Population differentiation (*F*_*ST*_) correlated with salinity (spawning-time and mean April estimates), but only once DE-RUGEN was excluded. For both salinity measures, this result remained consistent after correcting for oceanographic connectivity (60 loci and Her14 datasets), geographical distance (though only for Her14 locus), and temperature. Association tests between allele frequency and environmental variation showed that one particular Her14 allele (138) increased in frequency with increasing temperature and salinity, and decreased in frequency with increasing distance from the entrance to the Baltic and decreasing latitude. While the latter two variables are difficult to disentangle as they co-vary, the associations between genetic differentiation at locus Her14 with temperature and salinity were not caused by isolation by distance or oceanic connectivity, implying that the temperature and salinity correlations can not be explained by a demographic effect.

Altogether, our results indicate that both temperature and salinity differences between sites may contribute to the patterns of genetic differentiation, although the correlation between *F*_ST_ and April temperature was only significant with DE-RUGEN removed. In contrast, in the Jørgensen et al. (2005a) study, Rügen was found to be driving correlations between genetic divergence with salinity and temperature; when Rügen was removed, the analyses became non-significant. This is not an inconsistent result, as our correlations appear to be mainly driven by the Her14 locus, and it is clear that the Rügen site is divergent enough to strongly affect the results of broad scale correlation analyses. From a management perspective, identifying the spatial genetic structure, and environmental and oceanographic correlates is key. The causes of such structure and correlations may however not be limited to local adaptation and physical barriers to gene flow (oceanographic currents), as behavioural mechanisms (e.g. natal homing) and genetic incompatibilities among populations may also contribute.

### Management implications

The International Council for the Exploration of the Sea (ICES) currently divide herring in the Baltic region into five management units (Fig. 1A): Bothnian Bay (ICES fisheries subdivision SD31), Bothnian Sea (SD30), Baltic Sea (SD25-29 & 32 excluding the Gulf of Riga), Gulf of Riga (SD28.1), and Western Baltic together with spring-spawning herring in Kattegat and Skagerrak (SD22-24 and IIIa). While the Bothnian Bay and Gulf of Riga units seem to be relatively sustainable, the Baltic Sea and the western Baltic are experiencing stock declines and harvesting above the Maximum Sustainable Yield (ICES 2011).

Our results show conclusively that there is a general pattern of genetic differentiation between locations (*F*_ST_ and isolation by distance), as well as distinct genetic clusters of herring within the Baltic Sea. It is clear from our results, supported by the findings of previous studies (Bekkevold et al. 2005; Jørgensen et al. 2005a; Larsson et al. 2007), that the existing management units do not reflect the genetic divergence patterns in this species. Our genetic information indicates that DE-RUGEN, DK-FREDRIKSHAVN, SE-STROMSTAD, and LV-LIEPAJA form a distinct genetic cluster. Currently, DE-RUGEN, DK-FREDRIKSHAVN, SE-STROMSTAD are managed as a unit, whereas LV-LIEPAJA is not part of the SD22-24 and IIIa management unit. Additionally, we found that FI-VAASA is genetically distinct from its neighbouring regions, and that a population on the North coast of Estonia (EE-NARVANLAHTI) is genetically distinct.

We show that the mechanistically derived oceanographic connectivity provides partial explanation for the pattern of genetic differentiation in this area, and evidence was also found to indicate that patterns of genetic differentiation may be partially explained by site differences in mean temperature and salinity. Hence, it seems probable that the combined effects of oceanographic connectivity and environmental variables, as well as spawning behaviour together shape the genetic differentiation of herring in the Baltic Sea. Herring in the Baltic have also been shown to differ regionally in their weight, length, number of vertebrae, and number of pectoral fin rays (Parmanne 1990). This morphological variation may reflect phenotypic plasticity due to different environmental conditions during development (see Hulme 1995; Casini et al. 2010), but the possibility that it reflects adaptive genetic differentiation can also not currently be rejected.

We suggest that Baltic herring would be better managed on a finer geographical scale, taking both demographic isolation and local adaptation into account. In particular the large Baltic Sea unit (SD25-29 & 32 excluding the Gulf of Riga) presently comprises several distinct populations. Herring spawning in the southern Baltic along the German-Polish-Lithuanian-Latvian coast are connected oceanographically due to the anti-clockwise circulation pattern (Maslowski and Walczowski 2002), and possibly also genetically, as indicated by the clustering of the LV-LIEPAJA herring together with the DE-RUGEN herring. In contrast, the herring spawning along the Swedish coast (SE-KALMARSUND and SE-BLEKINGE) are more connected and similar genetically to spawning aggregations in the northern Baltic proper (FI-ECKERO). Subdivision of the central Baltic Sea unit would go some way to bringing the management units in line with the connectivity patterns, and also account better for potential adaptation to the local environment.

Rügen herring undertake feeding migrations to the Skagerrak and return to Rügen in spring for spawning (Biester 1979). Genetic mixed stock analyses show that the Rügen component is missing in the Skagerrak during a large part of the winter, suggesting that management of Eastern Baltic, Kattegat and Skagerrak spring-spawning herring should take more local stock dynamics into account (Bekkevold et al. 2011). Our findings of three distinct spawning populations within this area support this view.

Although we have shown genetic discontinuity, there are gaps in our geographical sampling regime, particularly the Gulf of Riga and the Polish coast, and we have not included Autumn spawning herring stocks in this study. More comprehensive sampling is required to hone our understanding of genetic differentiation in this region for a targeted study on Baltic management units. In particular this may help to resolve the issues surrounding the differentiation of Northern Baltic herring. However, we have shown that the Baltic herring do not represent a panmictic population, and in light of these findings, the management units in this region warrant reconsideration. Furthermore, our approach using a large number of gene-based molecular markers combined with oceanographic modelling and environmental data may prove useful for elucidating potential population units and subsequent management implications for other marine species. A recent study by Nielsen et al. (2012) has shown that such studies can also be invaluable for origin assignment in commercial fish.

## Data archiving statement

Data deposited in the Dryad repository: doi:10.5061/dryad.1qs20.

## Supporting Information

Table S1 Sampling information

Table S2 Primer multiplexes.

Table S3 Results from Lositan outlier tests.

Table S4 Results from BayeScan outlier tests.

Table S5 Allelic richness (*A*_R_) and expected heterozygosity (*H*_E_) for each population.

Table S6 F_IS_ and departure from Hardy Weinberg equilibrium.

Figure S1 Average F_IS_ estimates across all loci for each site.

Table S7 Locus-specific genetic diversity.

Table S8 Pairwise F_ST_ values between populations.

Figure S2 Structure results.

Figure S3 Bayesian clustering results from Structure for outlier loci.

Figure S4 Isolation by distance plots.

Figure S5 Plots of environmental data.

Table S9 Results from general linear models.

Table S10 Comparison between present study and previous studies, using the same nine microsatellites.
