# Supplementary material for: Oceanographic connectivity and environmental correlates of genetic structuring in Atlantic herring in the Baltic Sea
Source: Evol Appl. 2013 Feb 4;6(3):549–67. doi: 10.1111/eva.12042 (PMC3673481; doi:10.1111/eva.12042)
Supplement: Table S1 — Sampling information [file eva0006-0549-sd1.docx]

**Supporting Information 1: Sampling information**. Including site code, country, SD= ICES fisheries subdivision, latitude and longitude for each site are given to the accuracy provided by the collectors, collection date, approximate spawning time (one week on either side of the collection date), and *n* = number of individuals in final dataset.

| **Code** | **Country** | **SD** | **Latitude** | **Longitude** | **Collecting date** | **Approx. spawning time** | ***n*** |
| --- | --- | --- | --- | --- | --- | --- | --- |
| SE-STROMSTAD | Sweden | IIIa | 59° 5' 24" N | 11° 14' 9" E | 10 Apr 2009 | 03 Apr - 17 Apr | 47 |
| DK-FREDRIKSHAVN | Denmark | IIIa | 57° 18' 59" N | 10° 32' 15" E | 25 Apr - 5 May 2010 | 18 Apr - 12 May | 47 |
| DE-RUGEN | Germany | 24 | 54° 34' 28.28" N | 13° 27' 45.59" E | 15 Apr 2010 | 08 Apr - 22 Apr | 45 |
| LV-LIEPAJA | Latvia | 28 | 56° 47' 32.68" N | 21° 2' 43.83" E | 26 Apr 2010 | 19 Apr - 03 May | 47 |
| SE-BLEKINGE | Sweden | 25 | 55° 48' 9" N | 15° 7' 12" E | 05 Mar 2010 | 28 Apr - 12 May | 47 |
| EE-MUDASTE | Estonia | 29 | 59° 03' 6" N | 22° 28' 3" E | 03 May 2009 | 26 Apr - 10 May | 47 |
| SE-KALMARSUND | Sweden | 27 | 57° 23' 2" N | 16° 53' 2" E | 22 Feb 2010 | 15 Feb - 29 Feb | 47 |
| EE-NARVANAHTI | Estonia | 31 | 59° 25' N | 27° 33' E | 29 Apr 2009 | 22 Apr - 21 Jun | 46 |
| FI-BROMARV | Finland | 32 | 59° 54' 36" N | 23° 01' 24" E | 15 Jun 2009 | 08 Jun - 21 Jun | 47 |
| FI-ECKERO | Finland | 29 | 60° 11' 19.34'' N | 19° 36' 47.87'' E | 19 May 2009 | 12 May - 26 May | 47 |
| FI-VIROJOKI | Finland | 32 | 60° 30' 12" N | 27° 45' 30" E | 25 May 2009 | 18 May - 01 Jun | 43 |
| SE-UMEA | Sweden | 31 | 63° 24' 0.0'' N | 20° 19' 12.0'' E | 16 Jun 2009 | 09 Jun - 23 Jun | 47 |
| FI-SIMO | Finland | 31 | 65° 37' 29.96" N | 24° 52' 22.38" E | 23 Jun 2009 | 16 Jun - 29 Jun | 46 |
| FI-VAASA | Finland | 30 | 63° 6' 55.116" N | 21° 21' 10.529" E | 01 Jun 2009 | 25 May - 08 Jun | 47 |
| SE-LULEA | Sweden | 31 | 65° 25' N | 22° 32' E | 09 Jul 2010 | 02 Jul - 16 Jul | 44 |
