# Supplementary material for: Oceanographic connectivity and environmental correlates of genetic structuring in Atlantic herring in the Baltic Sea
Source: Evol Appl. 2013 Feb 4;6(3):549–67. doi: 10.1111/eva.12042 (PMC3673481; doi:10.1111/eva.12042)
Supplement: Table S2 — Primer multiplexes. [file eva0006-0549-sd2.doc]

**Supporting Information 2: Primer multiplexes. Showing the multiplex panel that each primer was used in, together with the dye used, and the approximate allele size range. Those marked in bold italics were removed during the study due to inconsistent and unreliable scoring, or the presence of null alleles.**

| **PANELS** | **FAM dye** | **Range** | **HEX dye** | **Range** | **TET dye** | **Range** |
| --- | --- | --- | --- | --- | --- | --- |
| **PAN1** | Her1 | 120-135 |  |  | Her18 | 160-180 |
|  | Her40 | 170-190 |  |  |  |  |
| **PAN2** | Her97 | 80-110 | Her101 | 80-100 | Her84 | 140-160 |
|  | Her67 | 150-170 |  |  |  |  |
| **PAN3** | Her22 | 110-130 | Her104 | 140-200 | Her102 | 90-110 |
|  | CPA114 | 200-250 |  |  | Her114 | 160-190 |
| **PAN4** | Her58 | 90-110 |  |  | Her132 | 130-150 |
|  | Her73 | 140-160 |  |  | ***Her54*** | 170-220 |
| **PAN5** | Her43 | 160-180 | Her14 | 120-140 | Her12 | 140-150 |
| **PAN6** | CHA1059 | 60-100 | Her59 | 240-250 | CPA101 | 170-300 |
|  | Her25 | 140-160 |  |  |  |  |
| **PAN7** | Her37 | 110-130 | Her20 | 90-110 | Her141 | 180-210 |
|  |  |  | CPA111 | 270-290 |  |  |
| **PAN8** | Her136 | 80-100 | Her41 | 110-120 | Her21 | 140-160 |
|  |  |  | Her62 | 165-170 |  |  |
| **PAN9** | Her124 | 95-120 |  |  | CPA104 | 170-250 |
| **PAN10** | Her142 | 80-110 | Her143 | 80-120 | Her36 | 160-180 |
| **PAN11** | CHA1017 | 150-200 | Her119 | 90-120 | CHA1020 | 150-250 |
|  | Her133 | 80-110 |  |  |  |  |
| **PAN12** | ***Her100*** | 120-140 | Her50 | 110-140 | Her63 | 100-130 |
|  |  |  |  |  | Her126 | 200-230 |
| **PAN13** | Her64 | 90-120 | Her77 | 160-180 | ***Her111*** | 130-140 |
| **PAN14** | Her130 | 100-130 | CPA105 | 150-200 |  |  |
| **PAN15** | CHA1202 | 80-140 | Her98 | 130-140 | Her117 | 110-120 |
| **PAN16** | CPA108 | 280-350 | CPA107 | 180-210 | CPA113 | 100-200 |
| **PAN17** | ***HER61*** | 100-130 | HER71 | 80-100 |  |  |
|  |  |  | CPA103 | 170-230 |  |  |
| **PAN18** | ***HER28*** | 80-110 | Her107 | 80-120 |  |  |
|  | ***CPA102*** | 150-230 |  |  |  |  |
| **PAN19** | HER118 | 140-150 | ***Her53*** | 110-130 |  |  |
|  |  |  | CPA112 | 230-280 |  |  |
| **PAN20** | ***CHA1005*** | 130-190 | Her140 | 110-140 |  |  |
| **PAN21** | Her109 | 120-140 | CHA1027 | 100-200 |  |  |
