# Supplementary material for: Oceanographic connectivity and environmental correlates of genetic structuring in Atlantic herring in the Baltic Sea
Source: Evol Appl. 2013 Feb 4;6(3):549–67. doi: 10.1111/eva.12042 (PMC3673481; doi:10.1111/eva.12042)
Supplement: Table S3 — Results from Lositan outlier tests. [file eva0006-0549-sd3.doc]

**Supporting Information 3: Results from Lositan outlier tests**. Expected heterozygosity (*H*_E_) and *F*_ST_ are given. The loci in bold were identified as 95% outliers, while those marked with asterix were identified as significant outliers at a false discovery rate of 0.05.

| **Locus** | ***H*_E_** | ***F*_ST_** | ***p(*Simul *F_ST_*< sample *F*_ST_)** |
| --- | --- | --- | --- |
| **CPA114** | **0.914** | **0.000** | **0.026** |
| CHA1202 | 0.769 | -0.001 | 0.118 |
| CPA103 | 0.871 | 0.001 | 0.129 |
| Her18 | 0.541 | -0.002 | 0.202 |
| Her118 | 0.340 | -0.002 | 0.227 |
| Her98 | 0.180 | -0.002 | 0.232 |
| Her133 | 0.678 | 0.001 | 0.262 |
| Her141 | 0.825 | 0.003 | 0.267 |
| Her21 | 0.263 | 0.000 | 0.301 |
| Her22 | 0.346 | 0.000 | 0.317 |
| Her132 | 0.497 | 0.000 | 0.321 |
| Her143 | 0.830 | 0.004 | 0.346 |
| CHA1059 | 0.668 | 0.003 | 0.346 |
| Her25 | 0.443 | 0.000 | 0.348 |
| Her20 | 0.422 | 0.001 | 0.357 |
| Her117 | 0.112 | 0.000 | 0.361 |
| Her102 | 0.240 | 0.002 | 0.392 |
| Her107 | 0.228 | 0.003 | 0.432 |
| Her119 | 0.756 | 0.004 | 0.453 |
| Her73 | 0.201 | 0.004 | 0.471 |
| Her140 | 0.820 | 0.005 | 0.478 |
| Her62 | 0.220 | 0.004 | 0.483 |
| Her40 | 0.143 | 0.003 | 0.492 |
| Her114 | 0.634 | 0.005 | 0.493 |
| Her64 | 0.566 | 0.005 | 0.506 |
| Her50 | 0.237 | 0.005 | 0.519 |
| Her59 | 0.520 | 0.005 | 0.528 |
| Her142 | 0.705 | 0.006 | 0.532 |
| Her58 | 0.315 | 0.005 | 0.536 |
| Her71 | 0.504 | 0.005 | 0.552 |
| Her97 | 0.629 | 0.006 | 0.569 |
| Her101 | 0.132 | 0.006 | 0.577 |
| CHA1027 | 0.931 | 0.005 | 0.585 |
| Her67 | 0.354 | 0.007 | 0.608 |
| Her12 | 0.493 | 0.007 | 0.610 |
| Her77 | 0.420 | 0.008 | 0.631 |
| Her1 | 0.533 | 0.008 | 0.652 |
| CPA101 | 0.913 | 0.006 | 0.657 |
| Her130 | 0.736 | 0.008 | 0.694 |
| CPA111 | 0.369 | 0.011 | 0.719 |
| Her104 | 0.885 | 0.008 | 0.735 |
| CHA1020 | 0.910 | 0.006 | 0.736 |
| CPA108 | 0.386 | 0.011 | 0.740 |
| Her43 | 0.158 | 0.011 | 0.752 |
| Her109 | 0.838 | 0.008 | 0.752 |
| Her136 | 0.305 | 0.011 | 0.754 |
| CPA113 | 0.935 | 0.006 | 0.761 |
| Her124 | 0.256 | 0.012 | 0.771 |
| CHA1017 | 0.815 | 0.009 | 0.779 |
| Her36 | 0.606 | 0.013 | 0.841 |
| CPA105 | 0.926 | 0.008 | 0.891 |
| Her126 | 0.550 | 0.018 | 0.913 |
| Her84 | 0.164 | 0.026 | 0.961 |
| **Her41** | **0.378** | **0.029** | **0.989** |
| **Her63** | **0.126** | **0.037** | **0.991** |
| **CPA112** | **0.762** | **0.020** | **0.993** |
| **Her37** | **0.075** | **0.042** | **0.995** |
| **CPA104** | **0.833** | **0.019** | **0.999** |
| **CPA107 *** | **0.555** | **0.032** | **1.000** |
| **Her14 *** | **0.572** | **0.080** | **1.000** |
