# Supplementary material for: Oceanographic connectivity and environmental correlates of genetic structuring in Atlantic herring in the Baltic Sea
Source: Evol Appl. 2013 Feb 4;6(3):549–67. doi: 10.1111/eva.12042 (PMC3673481; doi:10.1111/eva.12042)
Supplement: Table S4 — Results from BayeScan outlier tests. [file eva0006-0549-sd4.doc]

**Supporting Information 4: Results from BayeScan outlier tests. The posterior probability for the model including selection (*p*), the log10 of the Posterior Odds for the model including selection (log10(PO)), and the estimated alpha coefficient indicating the strength and direction of selection (alpha; positive values indicate positive selection, while negative values indicate putative balancing selection) are given for each locus. It should be noted that the power to detect loci under putative balancing selection is low. The loci in bold were identified as significant outliers under a false discovery rate of 0.05. The 20 loci in bold with the lowest *F*_ST_ values were identified as under putative balancing selection, while the two loci in bold with the highest *F*_ST_ values were identified as under positive selection.**

| **Locus** | ***p*** | **log10(PO)** | **alpha** | ***F*_ST_** |
| --- | --- | --- | --- | --- |
| **Her102** | **1.000** | **1000** | **-1.915** | **0.002** |
| **CPA103** | **1.000** | **1000** | **-1.838** | **0.002** |
| **CPA114** | **1.000** | **1000** | **-1.519** | **0.003** |
| **Her118** | **0.997** | **2.522** | **-1.563** | **0.003** |
| **Her107** | **1.000** | **3.398** | **-1.385** | **0.003** |
| **Her117** | **0.939** | **1.190** | **-1.509** | **0.003** |
| **Her119** | **0.999** | **3.222** | **-1.275** | **0.003** |
| **Her133** | **1.000** | **1000** | **-1.260** | **0.003** |
| **CHA1202** | **0.999** | **2.853** | **-1.256** | **0.003** |
| **Her20** | **0.961** | **1.389** | **-1.269** | **0.004** |
| **Her143** | **1.000** | **3.699** | **-1.180** | **0.004** |
| **Her25** | **0.904** | **0.974** | **-1.367** | **0.004** |
| **CPA101** | **1.000** | **1000** | **-1.079** | **0.004** |
| **Her140** | **0.969** | **1.492** | **-1.094** | **0.004** |
| **Her73** | **0.875** | **0.847** | **-1.166** | **0.004** |
| **Her141** | **0.970** | **1.513** | **-0.923** | **0.005** |
| **Her21** | **0.775** | **0.537** | **-1.016** | **0.005** |
| **CHA1020** | **0.968** | **1.481** | **-0.788** | **0.005** |
| **Her130** | **0.920** | **1.058** | **-0.736** | **0.005** |
| Her50 | 0.750 | 0.478 | -0.813 | 0.006 |
| CHA1059 | 0.651 | 0.270 | -0.617 | 0.007 |
| **Her104** | **0.837** | **0.711** | **-0.427** | **0.007** |
| CHA1027 | 0.749 | 0.475 | -0.379 | 0.007 |
| CPA113 | 0.692 | 0.351 | -0.363 | 0.008 |
| Her22 | 0.333 | -0.302 | -0.448 | 0.008 |
| Her98 | 0.337 | -0.294 | -0.418 | 0.008 |
| Her67 | 0.284 | -0.401 | -0.282 | 0.009 |
| Her59 | 0.232 | -0.519 | -0.285 | 0.009 |
| Her114 | 0.217 | -0.557 | -0.154 | 0.009 |
| Her12 | 0.130 | -0.825 | -0.109 | 0.010 |
| Her101 | 0.101 | -0.949 | -0.079 | 0.010 |
| Her40 | 0.093 | -0.989 | -0.070 | 0.010 |
| Her109 | 0.090 | -1.007 | -0.050 | 0.010 |
| CHA1017 | 0.075 | -1.094 | -0.039 | 0.010 |
| Her97 | 0.064 | -1.162 | -0.029 | 0.010 |
| Her58 | 0.069 | -1.127 | -0.032 | 0.010 |
| Her71 | 0.069 | -1.129 | -0.031 | 0.010 |
| CPA111 | 0.056 | -1.225 | -0.021 | 0.010 |
| CPA108 | 0.057 | -1.220 | -0.021 | 0.010 |
| Her77 | 0.050 | -1.277 | -0.016 | 0.010 |
| Her132 | 0.057 | -1.220 | -0.021 | 0.010 |
| Her1 | 0.061 | -1.187 | -0.022 | 0.010 |
| CPA104 | 0.027 | -1.553 | -0.007 | 0.010 |
| Her18 | 0.057 | -1.220 | -0.014 | 0.010 |
| Her43 | 0.047 | -1.305 | -0.010 | 0.010 |
| CPA105 | 0.024 | -1.617 | -0.004 | 0.010 |
| Her62 | 0.060 | -1.192 | -0.018 | 0.010 |
| Her64 | 0.042 | -1.362 | -0.008 | 0.010 |
| Her142 | 0.028 | -1.537 | -0.002 | 0.010 |
| Her84 | 0.047 | -1.303 | -0.004 | 0.011 |
| Her36 | 0.032 | -1.475 | 0.000 | 0.011 |
| Her124 | 0.051 | -1.271 | 0.001 | 0.011 |
| Her136 | 0.050 | -1.282 | 0.006 | 0.011 |
| CPA112 | 0.104 | -0.934 | 0.037 | 0.011 |
| Her37 | 0.071 | -1.119 | 0.034 | 0.011 |
| Her126 | 0.119 | -0.869 | 0.061 | 0.011 |
| Her41 | 0.209 | -0.578 | 0.172 | 0.014 |
| Her63 | 0.380 | -0.212 | 0.438 | 0.020 |
| **CPA107** | **0.958** | **1.362** | **1.077** | **0.032** |
| **Her14** | **1.000** | **1000** | **1.517** | **0.046** |
