# Supplementary material for: Oceanographic connectivity and environmental correlates of genetic structuring in Atlantic herring in the Baltic Sea
Source: Evol Appl. 2013 Feb 4;6(3):549–67. doi: 10.1111/eva.12042 (PMC3673481; doi:10.1111/eva.12042)
Supplement: Table S5 — Allelic richness (AR) and expected heterozygosity (HE) for each population. [file eva0006-0549-sd5.doc]

**Supporting Information 5: Allelic richness (*A*_R_) and expected heterozygosity (*H*_E_) for each population. Different datasets of loci were used: all 60 loci, 59 loci (excluding Her14), and locus Her14, and the mean, minimum, and maximum are given for each measure for each dataset.**

|  | ***A*_R_** | | | ***H*_E_** | | |
| --- | --- | --- | --- | --- | --- | --- |
| **Population** | **60 loci** | **59 loci** | **Her14** | **60 loci** | **59 loci** | **Her14** |
| SE-STROMSTAD | 5.713 | 5.741 | 4.093 | 0.548 | 0.545 | 0.686 |
| DK-FREDRIKSHAVN | 5.751 | 5.780 | 4.001 | 0.551 | 0.549 | 0.676 |
| DE-RUGEN | 5.169 | 5.206 | 2.998 | 0.522 | 0.522 | 0.539 |
| LV-LIEPAJA | 5.518 | 5.545 | 3.907 | 0.532 | 0.530 | 0.603 |
| EE-MUDASTE | 5.416 | 5.438 | 4.156 | 0.503 | 0.504 | 0.475 |
| SE-BLEKINGE | 5.557 | 5.588 | 3.769 | 0.521 | 0.521 | 0.534 |
| SE-KALMARSUND | 5.484 | 5.516 | 3.609 | 0.515 | 0.516 | 0.467 |
| FI-BROMARV | 5.536 | 5.571 | 3.492 | 0.519 | 0.519 | 0.510 |
| EE-NARVANLAHTI | 4.981 | 5.008 | 3.388 | 0.489 | 0.489 | 0.464 |
| FI-VIROJOKI | 5.608 | 5.637 | 3.860 | 0.526 | 0.526 | 0.485 |
| FI-ECKERO | 5.591 | 5.630 | 3.304 | 0.516 | 0.517 | 0.488 |
| FI-VAASA | 4.804 | 4.844 | 2.410 | 0.467 | 0.467 | 0.479 |
| SE-UMEA | 5.608 | 5.640 | 3.711 | 0.517 | 0.518 | 0.492 |
| FI-SIMO | 5.451 | 5.488 | 3.281 | 0.523 | 0.524 | 0.482 |
| SE-LULEA | 5.598 | 5.642 | 3.001 | 0.520 | 0.521 | 0.516 |
| **Mean** | 5.452 | 5.485 | 3.532 | 0.518 | 0.518 | 0.526 |
| **Min** | 4.804 | 4.844 | 2.410 | 0.467 | 0.467 | 0.464 |
| **Max** | 5.751 | 5.780 | 4.156 | 0.551 | 0.549 | 0.686 |
