# Supplementary material for: Oceanographic connectivity and environmental correlates of genetic structuring in Atlantic herring in the Baltic Sea
Source: Evol Appl. 2013 Feb 4;6(3):549–67. doi: 10.1111/eva.12042 (PMC3673481; doi:10.1111/eva.12042)
Supplement: Table S6 — FIS and departure from Hardy Weinberg equilibrium. [file eva0006-0549-sd6.doc]

**Supporting Information 6: *F*_IS_ and departure from Hardy Weinberg equilibrium. The p-values refer to tests for heterozygote deficiency, those that were significant after Bonferroni correction are given in bold.**

| **Locus** | **SE-STROMSTAD** | **DK-FREDRIK.** | **DE-RUGEN** | **LV-LIEPAJA** | **EE-MUDASTE** | **SE-BLEKINGE** | **SE-KALMARSUND** | **FI-BROMARV** | **EE-NARVANLAHTI** | **FI-VIROJOKI** | **FI-ECKERO** | **FI-VAASA** | **SE-UMEA** | **FI-SIMO** | **SE-LULEA** |
| --- | --- | --- | --- | --- | --- | --- | --- | --- | --- | --- | --- | --- | --- | --- | --- |
| **Her1** | -0.090 | -0.020 | -0.371 | 0.128 | -0.326 | 0.167 | -0.539 | -0.040 | -0.665 | 0.049 | 0.016 | -0.826 | -0.210 | -0.170 | -0.376 |
| **Her12** | 0.000 | -0.098 | -0.250 | -0.218 | -0.335 | 0.024 | -0.241 | 0.005 | -0.414 | -0.086 | 0.033 | -0.439 | -0.052 | -0.012 | -0.174 |
| **Her14** | 0.255* | -0.092 | 0.001 | 0.083 | 0.098 | 0.084 | 0.053 | 0.250* | -0.078* | -0.080 | -0.025 | -0.552 | 0.169 | -0.014 | 0.216 |
| **Her18** | -0.015 | 0.210* | -0.240 | -0.065 | -0.087 | 0.012 | -0.094 | -0.118 | -0.210 | -0.180 | -0.158 | -0.608 | -0.160 | 0.014 | -0.128 |
| **Her20** | 0.027 | 0.100 | -0.145 | 0.078 | -0.063 | 0.088 | 0.057 | -0.011 | -0.026 | 0.196* | 0.042 | -0.137 | 0.204* | 0.027 | 0.173* |
| **Her21** | -0.099 | -0.009 | -0.106 | -0.077 | -0.132 | -0.061 | -0.108 | 0.042 | -0.095 | -0.078 | -0.089 | -0.167 | -0.101 | 0.094 | -0.082 |
| **Her22** | 0.045 | -0.108 | -0.123 | -0.143 | -0.194 | -0.097 | 0.099 | 0.118 | -0.161 | 0.224 | -0.143 | -0.227 | 0.061 | -0.032 | -0.071 |
| **Her25** | -0.143 | -0.182 | -0.216 | 0.138 | -0.055 | 0.038 | 0.125 | -0.015 | -0.210 | 0.146** | 0.108 | -0.401 | 0.085 | 0.238 | 0.304** |
| **Her36** | -0.001 | -0.091 | 0.024 | 0.262** | -0.174 | 0.087 | 0.072 | 0.012 | -0.265 | 0.168* | 0.024 | -0.209 | 0.118* | -0.009 | 0.107 |
| **Her37** | 0.233 | 0.338* | -0.023 | -0.129 | -0.022 | -0.006 | -0.047 | -0.012 | -0.148 | -0.024 | -0.011 | -0.034 | -0.015 | 0.233 | -0.024 |
| **Her40** | -0.046 | 0.125 | -0.250 | -0.067 | -0.098 | 0.297 | -0.227 | -0.073 | -0.008 | -0.085 | -0.126 | -0.150 | -0.025 | -0.047 | -0.079 |
| **Her41** | 0.014 | -0.095 | -0.078 | 0.121 | -0.040 | 0.088** | -0.015 | -0.075 | -0.038 | -0.201 | -0.199 | -0.074 | 0.021 | 0.049 | -0.080 |
| **Her43** | 0.041 | -0.062 | -0.132 | -0.009 | -0.029 | 0.309 | -0.030 | -0.051 | -0.063 | 0.137 | -0.070 | -0.046 | 0.309 | -0.006 | -0.059 |
| **Her50** | -0.098 | 0.028 | -0.104 | 0.037 | -0.036 | -0.009* | 0.045 | -0.071 | 0.081 | -0.057 | -0.036 | -0.133 | -0.066 | 0.143 | 0.010 |
| **Her58** | 0.498* | 0.460* | -0.189 | 0.476*** | 0.409** | 0.190 | -0.103 | 0.239* | -0.394 | 0.376* | 0.344* | -0.729 | 0.499*** | 0.310* | 0.200 |
| **Her59** | 0.269* | -0.077 | -0.222 | 0.103 | -0.234 | 0.182 | 0.159 | 0.237 | -0.008 | 0.120 | -0.093 | -0.136 | 0.228 | 0.057 | -0.060 |
| **Her62** | 0.107 | -0.122 | -0.065 | 0.647*** | -0.148 | -0.128 | -0.258 | 0.399* | -0.023 | -0.093 | -0.071 | -0.046 | 0.179 | -0.084 | -0.049 |
| **Her63** | 0.186 | -0.206 | -0.021 | 0.093 | -0.034 | -0.058 | 0.011 | -0.058 | -0.194 | 0.373 | -0.023 | -0.381 | -0.061 | -0.077 | -0.024 |
| **Her64** | 0.023 | 0.042 | -0.147 | 0.012* | -0.038 | -0.119 | -0.191 | 0.088 | -0.156 | 0.012 | -0.022 | -0.052 | -0.101 | -0.136 | -0.051 |
| **Her67** | -0.116 | 0.141 | -0.011 | -0.100 | 0.014 | 0.048 | 0.082 | 0.022 | -0.223 | -0.083 | -0.194 | -0.787 | 0.131 | 0.019 | 0.097 |
| **Her71** | 0.166 | -0.011 | -0.065 | -0.089 | -0.058 | 0.020 | -0.060 | 0.091 | -0.140 | 0.109 | -0.063 | -0.058 | 0.026 | 0.037 | -0.184 |
| **Her73** | -0.012 | -0.085 | -0.098 | 0.168 | -0.058 | -0.055 | -0.034 | 0.410* | -0.134 | -0.074 | 0.076 | -0.172 | 0.069 | 0.204 | 0.059 |
| **Her77** | -0.095 | 0.238 | -0.116 | -0.084 | -0.127 | -0.007 | -0.138 | 0.131* | 0.373 | 0.099 | -0.128 | -0.025 | 0.100 | -0.052 | -0.121 |
| **Her84** | -0.058 | -0.108 | 0.079 | 0.095 | -0.051 | -0.046 | -0.099 | -0.059 | -0.090 | 0.117 | -0.052 | -0.139 | -0.036 | -0.059 | -0.027 |
| **Her97** | -0.046 | -0.066 | -0.040 | -0.058 | -0.005 | 0.024 | -0.059 | -0.044 | -0.031 | 0.065 | 0.092* | -0.110 | -0.011 | 0.109 | 0.053 |
| **Her98** | -0.123 | -0.078 | -0.144 | -0.046 | -0.061 | -0.056 | 0.266 | 0.086 | -0.024 | -0.076 | -0.059 | -0.040 | -0.113 | 0.087 | -0.077 |
| **Her101** | -0.075 | -0.031 | -0.097 | 0.029 | -0.046 | 0.338* | 0.199 | -0.034 | -0.112 | -0.037 | -0.099 | -0.082 | -0.064 | 0.245 | -0.062 |
| **Her102** | 0.032 | 0.061 | 0.081 | -0.003 | -0.067 | -0.059 | 0.129 | 0.056 | 0.138 | -0.149 | -0.034 | -0.093 | 0.218 | -0.078 | -0.071 |
| **Her104** | 0.061 | -0.002 | -0.034 | 0.220 | 0.107 | 0.033 | **0.158***** | 0.103 | -0.045 | -0.010 | -0.024 | -0.062 | **0.118***** | 0.160 | **0.119***** |
| **Her107** | -0.064 | -0.050 | -0.048 | 0.006* | 0.219* | -0.041 | 0.021 | -0.068 | 0.023 | 0.148* | 0.008 | 0.013 | 0.138* | -0.080 | -0.044 |
| **Her108** | -0.040* | -0.088 | -0.172 | 0.061 | 0.130*** | -0.068 | -0.036* | -0.008 | -0.130 | -0.057 | 0.101 | 0.017 | 0.153* | 0.058 | -0.001 |
| **Her114** | -0.025 | 0.179 | -0.062 | -0.047 | 0.120 | -0.089 | 0.131 | 0.263*** | -0.064 | 0.030 | 0.101* | -0.029 | 0.662* | 0.123* | **0.166***** |
| **Her117** | -0.095 | 0.237 | -0.104 | 0.365 | -0.084 | -0.022 | 0.170 | -0.036 | -0.123 | 0.234 | -0.022 | -0.109 | 0.010* | 0.164 | -0.049 |
| **Her118** | 0.038 | -0.017 | -0.112 | 0.147*** | -0.091 | 0.003 | -0.117 | -0.179 | 0.087 | 0.193 | 0.008 | 0.024 | -0.012 | 0.136 | -0.003 |
| **Her119** | -0.065 | 0.008 | -0.135 | 0.103 | 0.137 | 0.079 | -0.215 | 0.083* | -0.171 | 0.041* | 0.230* | 0.189* | -0.117 | 0.061 | 0.037 |
| **Her124** | -0.025 | 0.050 | 0.018 | 0.107 | -0.078 | -0.112 | 0.050 | -0.119 | -0.331 | -0.048 | -0.054 | -0.591 | 0.190 | 0.045 | -0.056 |
| **Her126** | -0.173 | -0.017 | -0.273 | -0.045** | -0.256 | -0.132 | -0.084 | 0.043 | -0.139 | 0.120 | 0.030 | -0.190 | -0.088 | 0.117 | 0.042 |
| **Her130** | **0.058***** | -0.012 | -0.346 | 0.052 | -0.079 | 0.110* | -0.052 | 0.172** | -0.260 | 0.047 | -0.111 | -0.500 | -0.055 | -0.099* | 0.039 |
| **Her132** | -0.087 | -0.055 | -0.055 | -0.096 | 0.024 | -0.294 | -0.199 | 0.128 | 0.039 | 0.206 | 0.245*** | 0.000 | 0.059 | -0.051 | 0.003 |
| **Her133** | -0.023 | -0.045 | -0.049 | 0.002 | 0.027 | -0.044 | 0.107 | -0.048 | -0.083 | 0.179* | -0.043 | -0.086 | -0.071 | -0.007 | -0.173 |
| **Her136** | 0.031 | -0.033 | -0.061 | -0.024 | -0.150 | -0.070 | 0.084 | 0.081 | 0.109 | 0.358** | -0.021 | 0.074 | -0.029 | -0.075 | 0.045 |
| **Her140** | -0.028 | 0.090 | -0.120 | -0.063 | 0.058* | -0.056 | 0.027 | 0.030 | 0.053* | 0.033 | 0.174** | -0.160 | 0.038 | 0.010 | -0.008 |
| **Her141** | -0.018 | 0.079* | -0.157 | 0.160 | -0.025 | 0.108 | -0.009** | -0.044 | -0.319 | 0.055 | 0.084* | -0.341 | -0.005 | 0.034 | 0.059 |
| **Her142** | -0.032 | 0.031* | -0.097 | **0.173***** | 0.127 | -0.054 | -0.009 | -0.124 | 0.058 | -0.060 | 0.102 | -0.069 | -0.023 | 0.088 | -0.062 |
| **Her143** | 0.025 | 0.061 | -0.004 | **0.049***** | 0.100* | 0.021 | 0.176 | 0.051 | 0.202** | -0.007 | 0.037 | 0.145 | 0.025 | -0.021 | -0.069 |
| **CHA1017** | 0.219* | 0.042** | -0.015 | 0.121*** | 0.250** | 0.125* | 0.037 | 0.099 | 0.087 | 0.008 | 0.025 | 0.118 | 0.026 | -0.044 | 0.054 |
| **CHA1020** | 0.011 | -0.046 | -0.003 | 0.065* | -0.067 | 0.047** | 0.119 | 0.018 | 0.190 | 0.000 | 0.070 | 0.152 | 0.107 | -0.047 | 0.045 |
| **CHA1027** | 0.094 | 0.029 | -0.023 | 0.325 | 0.039 | -0.020 | 0.089 | -0.046 | 0.102** | -0.004 | 0.019* | -0.158** | 0.187 | 0.046 | 0.098* |
| **CHA1059** | 0.007 | 0.083 | -0.032 | 0.036*** | 0.121 | 0.005 | 0.007* | 0.037 | -0.031 | **0.191***** | 0.154* | -0.178 | 0.004** | 0.115* | 0.063 |
| **CHA1202** | 0.171* | -0.100 | -0.073 | 0.056 | 0.040 | 0.092 | 0.089 | 0.015 | -0.058 | 0.011 | 0.097** | -0.161 | 0.058 | 0.035 | -0.099 |
| **CPA101** | 0.012 | 0.032 | 0.067 | -0.063* | 0.057 | -0.068 | 0.028 | 0.049 | -0.073* | -0.029 | 0.035 | 0.112 | 0.101** | 0.151 | 0.055 |
| **CPA103** | 0.026 | 0.035 | 0.023 | 0.022 | 0.076* | 0.076 | 0.226*** | -0.003 | -0.050 | 0.063 | 0.023 | -0.082 | 0.069** | 0.042* | 0.052 |
| **CPA104** | 0.126 | 0.012 | 0.047 | 0.077 | -0.005 | 0.043 | -0.022* | -0.031 | 0.244 | -0.024 | 0.030 | 0.154 | 0.052* | 0.103 | 0.024 |
| **CPA105** | 0.031 | 0.113* | -0.170 | 0.102* | 0.023 | **0.163***** | -0.094** | 0.038 | **-0.319***** | **0.146***** | 0.103** | -0.820* | 0.144 | 0.151* | 0.174* |
| **CPA107** | 0.266*** | -0.086 | -0.084 | -0.152 | -0.104 | 0.112* | -0.052 | -0.070 | 0.005 | -0.171 | -0.127 | -0.106 | 0.110* | -0.024 | -0.051 |
| **CPA108** | -0.078 | 0.045 | 0.024 | -0.038 | -0.115 | -0.050 | -0.057 | 0.097* | -0.103 | -0.106 | -0.154 | -0.177 | -0.008* | -0.113 | 0.054 |
| **CPA111** | -0.022 | 0.130 | -0.042 | 0.099 | -0.214 | -0.194 | 0.004 | -0.067 | -0.249 | 0.025 | -0.139 | -0.258 | 0.079 | -0.051 | 0.037 |
| **CPA112** | 0.021 | 0.031 | 0.025 | -0.072 | -0.063 | 0.161** | 0.087 | -0.100 | 0.018 | 0.041 | -0.047 | -0.041 | 0.095 | 0.023 | 0.012 |
| **CPA113** | -0.003 | -0.020 | 0.130 | 0.034 | 0.000 | 0.047 | 0.018 | 0.070 | 0.168 | 0.020 | 0.050 | 0.092 | 0.125* | 0.063 | 0.049 |
| **CPA114** | 0.062 | 0.126* | 0.018* | 0.110 | -0.001 | 0.028 | 0.020* | 0.073 | **0.113***** | 0.026 | 0.010 | -0.107* | 0.102* | 0.023 | 0.010 |
| **All** | **0.027**** | 0.018* | -0.073 | **0.056***** | -0.009* | **0.025***** | **0.007***** | **0.034***** | -0.058** | **0.040***** | **0.023***** | -0.161 | **0.058***** | 0.035** | **0.014**** |

*p<0.05; **p<0.01; *** p<0.001
