# Supplementary material for: Oceanographic connectivity and environmental correlates of genetic structuring in Atlantic herring in the Baltic Sea
Source: Evol Appl. 2013 Feb 4;6(3):549–67. doi: 10.1111/eva.12042 (PMC3673481; doi:10.1111/eva.12042)
Supplement: Figure S1 — Average FIS estimates across all loci for each site. [file eva0006-0549-sd7.docx]

**Supporting Information 7: Average *F*_IS_ estimates across all loci for each site.** The average *F*_IS_ values for each site are shown by horizontal lines, while the 95% confidence intervals are shown by dotted line bounded by the upper and lower circles.

**
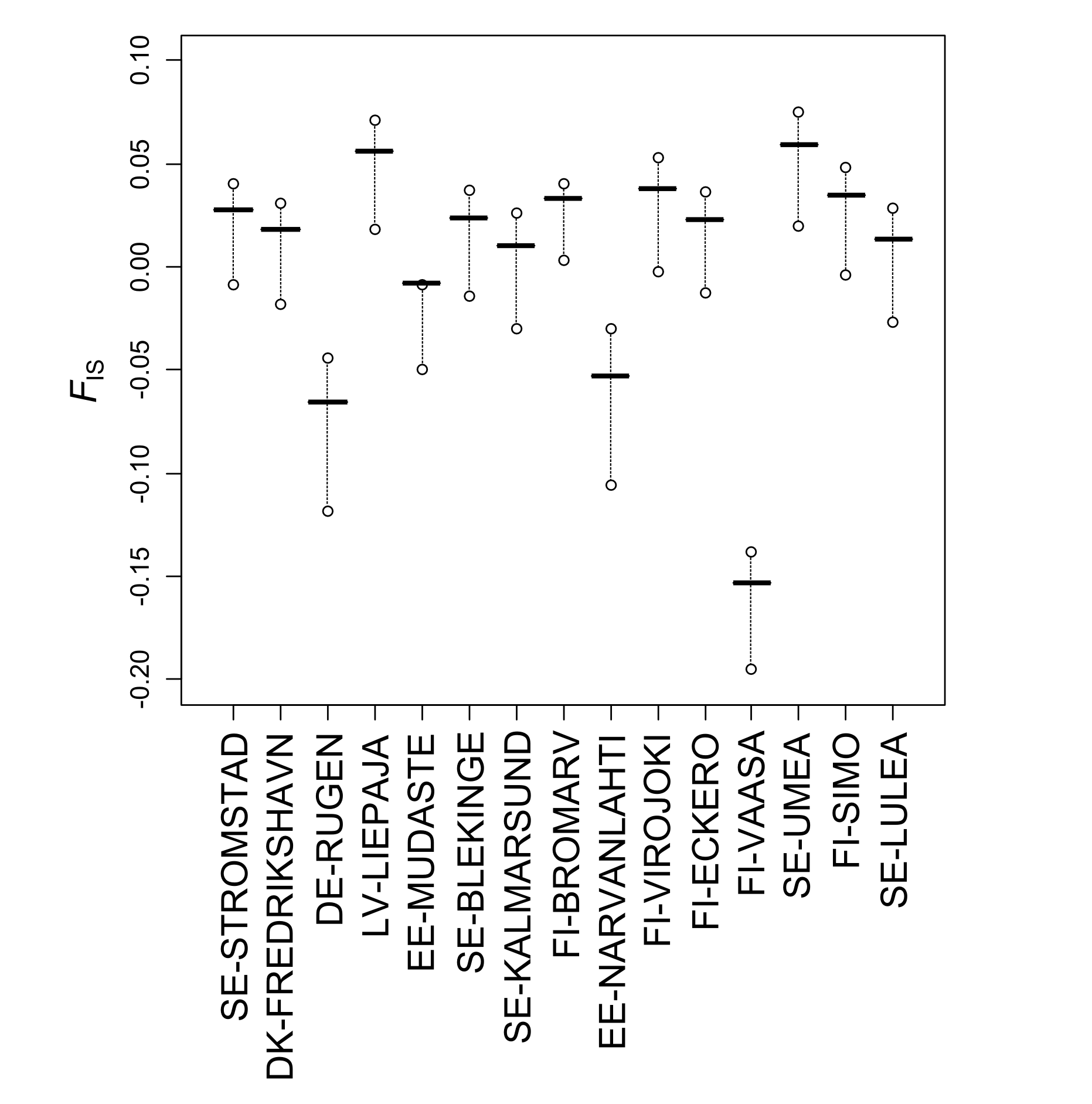
**
