# Supplementary material for: Oceanographic connectivity and environmental correlates of genetic structuring in Atlantic herring in the Baltic Sea
Source: Evol Appl. 2013 Feb 4;6(3):549–67. doi: 10.1111/eva.12042 (PMC3673481; doi:10.1111/eva.12042)
Supplement: Table S7 — Locus-specific genetic diversity. [file eva0006-0549-sd8.docx]

**Supporting Information 8: Locus-specific genetic diversity.** Shown are *F*_ST_ and associated *p*-value obtained using the exact G-test in Genepop 4.1.0 (*F*_ST_-values with *p*<0.05 are in bold), expected heterozygosity (*H*_E_) and allelic richness (*A*_R_).

| Locus | *F*_ST_ | *p* | *H*_E_ | *A*_R_ |
| --- | --- | --- | --- | --- |
| Her1 | 0.010 | 0.061 | 0.528 | 2.956 |
| Her101 | 0.006 | 0.046 | 0.132 | 2.241 |
| Her102 | 0.002 | 0.913 | 0.239 | 3.242 |
| Her104 | 0.007 | 0.000 | 0.879 | 12.868 |
| Her107 | 0.003 | 0.051 | 0.228 | 4.394 |
| Her109 | 0.007 | 0.000 | 0.831 | 8.035 |
| Her114 | 0.004 | 0.014 | 0.631 | 5.932 |
| Her117 | 0.000 | 0.463 | 0.112 | 1.990 |
| Her118 | -0.002 | 0.909 | 0.341 | 4.383 |
| Her119 | 0.003 | 0.154 | 0.754 | 6.524 |
| Her12 | 0.007 | 0.084 | 0.489 | 3.037 |
| Her124 | 0.013 | 0.000 | 0.253 | 3.162 |
| Her126 | 0.018 | 0.000 | 0.539 | 3.282 |
| Her130 | 0.008 | 0.000 | 0.730 | 9.315 |
| Her132 | 0.001 | 0.250 | 0.496 | 2.694 |
| Her133 | 0.002 | 0.015 | 0.677 | 7.888 |
| Her136 | 0.012 | 0.000 | 0.302 | 2.921 |
| Her14 | 0.075 | 0.000 | 0.526 | 3.852 |
| Her140 | 0.005 | 0.011 | 0.816 | 7.237 |
| Her141 | 0.003 | 0.025 | 0.823 | 8.611 |
| Her142 | 0.006 | 0.000 | 0.700 | 6.624 |
| Her143 | 0.003 | 0.005 | 0.827 | 9.335 |
| Her18 | -0.001 | 0.004 | 0.541 | 3.022 |
| Her20 | 0.000 | 0.478 | 0.422 | 4.455 |
| Her21 | 0.000 | 0.645 | 0.263 | 3.238 |
| Her22 | 0.001 | 0.602 | 0.346 | 2.415 |
| Her25 | 0.001 | 0.460 | 0.443 | 2.559 |
| Her36 | 0.012 | 0.000 | 0.598 | 5.001 |
| Her37 | 0.042 | 0.000 | 0.072 | 1.828 |
| Her40 | 0.004 | 0.287 | 0.142 | 2.013 |
| Her41 | 0.030 | 0.000 | 0.367 | 2.264 |
| Her43 | 0.010 | 0.000 | 0.156 | 2.742 |
| Her50 | 0.005 | 0.108 | 0.236 | 3.621 |
| Her58 | 0.002 | 0.002 | 0.314 | 3.109 |
| Her59 | 0.006 | 0.315 | 0.517 | 2.639 |
| Her62 | 0.004 | 0.182 | 0.219 | 2.170 |
| Her63 | 0.037 | 0.000 | 0.121 | 1.978 |
| Her64 | 0.005 | 0.009 | 0.562 | 3.509 |
| Her67 | 0.008 | 0.034 | 0.351 | 3.722 |
| Her71 | 0.006 | 0.122 | 0.500 | 2.649 |
| Her73 | 0.003 | 0.226 | 0.201 | 3.009 |
| Her77 | 0.008 | 0.000 | 0.417 | 5.477 |
| Her84 | 0.026 | 0.000 | 0.160 | 2.307 |
| Her97 | 0.006 | 0.002 | 0.625 | 5.880 |
| Her98 | -0.002 | 0.650 | 0.180 | 2.462 |
| CHA1017 | 0.008 | 0.000 | 0.808 | 7.315 |
| CHA1020 | 0.005 | 0.006 | 0.905 | 12.401 |
| CHA1027 | 0.004 | 0.000 | 0.927 | 14.531 |
| CHA1059 | 0.002 | 0.054 | 0.667 | 6.422 |
| CHA1202 | -0.001 | 0.333 | 0.770 | 7.785 |
| CPA101 | 0.005 | 0.028 | 0.908 | 12.787 |
| CPA103 | 0.000 | 0.537 | 0.871 | 9.602 |
| CPA104 | 0.018 | 0.000 | 0.817 | 10.886 |
| CPA105 | 0.006 | 0.000 | 0.920 | 13.558 |
| CPA107 | 0.035 | 0.000 | 0.537 | 3.373 |
| CPA108 | 0.011 | 0.001 | 0.382 | 4.535 |
| CPA111 | 0.011 | 0.003 | 0.365 | 3.914 |
| CPA112 | 0.020 | 0.000 | 0.746 | 8.990 |
| CPA113 | 0.006 | 0.000 | 0.929 | 14.025 |
| CPA114 | -0.001 | 0.351 | 0.915 | 12.117 |
| All: | 0.008 | 0.000 | 0.518 | 5.581 |
