# Supplementary material for: Oceanographic connectivity and environmental correlates of genetic structuring in Atlantic herring in the Baltic Sea
Source: Evol Appl. 2013 Feb 4;6(3):549–67. doi: 10.1111/eva.12042 (PMC3673481; doi:10.1111/eva.12042)
Supplement: Table S8 — Pairwise FST values between populations. [file eva0006-0549-sd9.docx]

**Supporting Information 9: Pairwise *F*_ST_ values between populations.** *F*_ST_ values are shown below the diagonal, and associated probability values are shown above the diagonal, calculated using the exact G-test in Genepop 4.1.0. Those marked in bold were significant at a False Discovery Rate of *q*<0.05. Three tables are given showing the *F*_ST_ and *p*-values for (i) all 60 loci, (ii) 59 loci (excluding Her14), and (iii) locus Her14.

1. All 60 loci

|  | SE-STROMSTAD | DK-FREDRIKSHAVN | DE-RUGEN | LV-LIEPAJA | EE-MUDASTE | SE-BLEKINGE | SE-KALMARSUND | FI-BROMARV | EE-NARVANLAHTI | FI-VIROJOKI | FI-ECKERO | FI-VAASA | SE-UMEA | FI-SIMO | SE-LULEA |
| --- | --- | --- | --- | --- | --- | --- | --- | --- | --- | --- | --- | --- | --- | --- | --- |
| SE-STROMSTAD | - | **0.022** | **0.000** | **0.000** | **0.000** | **0.000** | **0.000** | **0.000** | **0.000** | **0.000** | **0.000** | **0.000** | **0.000** | **0.000** | **0.000** |
| DK-FREDRIKSHAVN | 0.001 | - | **0.000** | **0.000** | **0.000** | **0.000** | **0.000** | **0.000** | **0.000** | **0.000** | **0.000** | **0.000** | **0.000** | **0.000** | **0.000** |
| DE-RUGEN | 0.014 | 0.011 | - | **0.000** | **0.000** | **0.000** | **0.000** | **0.000** | **0.000** | **0.000** | **0.000** | **0.000** | **0.000** | **0.000** | **0.000** |
| LV-LIEPAJA | 0.008 | 0.010 | 0.016 | - | **0.000** | **0.000** | **0.000** | **0.000** | **0.000** | **0.000** | **0.000** | **0.000** | **0.000** | **0.000** | **0.000** |
| EE-MUDASTE | 0.009 | 0.009 | 0.021 | 0.005 | - | 0.435 | 0.458 | 0.306 | **0.000** | 0.522 | **0.012** | **0.000** | 0.055 | **0.036** | **0.021** |
| SE-BLEKINGE | 0.010 | 0.011 | 0.019 | 0.005 | 0.000 | - | 0.591 | 0.529 | **0.000** | 0.315 | **0.014** | **0.000** | 0.311 | 0.170 | **0.012** |
| SE-KALMARSUND | 0.008 | 0.009 | 0.019 | 0.006 | 0.000 | -0.001 | - | 0.174 | **0.000** | 0.225 | **0.002** | **0.000** | 0.280 | **0.021** | 0.057 |
| FI-BROMARV | 0.008 | 0.009 | 0.020 | 0.006 | 0.003 | 0.000 | 0.001 | - | **0.000** | 0.895 | 0.101 | **0.003** | 0.770 | 0.254 | 0.155 |
| EE-NARVANLAHTI | 0.017 | 0.020 | 0.031 | 0.012 | 0.008 | 0.006 | 0.008 | 0.006 | - | **0.000** | **0.000** | **0.000** | **0.000** | **0.000** | **0.000** |
| FI-VIROJOKI | 0.009 | 0.008 | 0.021 | 0.005 | 0.001 | 0.002 | -0.001 | 0.000 | 0.010 | - | 0.137 | **0.000** | 0.800 | **0.031** | 0.374 |
| FI-ECKERO | 0.013 | 0.012 | 0.025 | 0.007 | 0.003 | 0.001 | 0.003 | 0.002 | 0.009 | 0.002 | - | **0.000** | **0.020** | **0.000** | 0.048 |
| FI-VAASA | 0.022 | 0.025 | 0.033 | 0.016 | 0.010 | 0.008 | 0.009 | 0.006 | 0.009 | 0.011 | 0.009 | - | **0.002** | **0.000** | **0.000** |
| SE-UMEA | 0.009 | 0.009 | 0.022 | 0.004 | 0.002 | 0.001 | -0.001 | -0.001 | 0.007 | -0.001 | 0.002 | 0.007 | - | 0.104 | 0.217 |
| FI-SIMO | 0.009 | 0.012 | 0.022 | 0.007 | 0.004 | 0.002 | 0.002 | 0.003 | 0.009 | 0.004 | 0.006 | 0.010 | 0.001 | - | **0.015** |
| SE-LULEA | 0.005 | 0.009 | 0.021 | 0.006 | 0.003 | 0.002 | 0.002 | 0.004 | 0.010 | 0.001 | 0.002 | 0.012 | 0.001 | 0.003 | - |

1. 59 loci (excluding Her14)

| FST\P | SE-STROMSTAD | DK-FREDRIKSH. | DE-RUGEN | LV-LIEPAJA | EE-MUDASTE | SE-BLEKINGE | SE-KALMARSUND | FI-BROMARV | EE-NARVANLAHTI | FI-VIROJOKI | FI-ECKERO | FI-VAASA | SE-UMEA | FI-SIMO | SE-LULEA |
| --- | --- | --- | --- | --- | --- | --- | --- | --- | --- | --- | --- | --- | --- | --- | --- |
| SE-STROMSTAD | - | **0.018** | **0.000** | **0.000** | **0.000** | **0.000** | **0.000** | **0.000** | **0.000** | **0.000** | **0.000** | **0.000** | **0.000** | **0.000** | **0.000** |
| DK-FREDRIKSHAVN | 0.001 | - | **0.000** | **0.000** | **0.000** | **0.000** | **0.000** | **0.000** | **0.000** | **0.000** | **0.000** | **0.000** | **0.000** | **0.000** | **0.000** |
| DE-RUGEN | 0.010 | 0.009 | - | **0.000** | **0.000** | **0.000** | **0.000** | **0.000** | **0.000** | **0.000** | **0.000** | **0.000** | **0.000** | **0.000** | **0.000** |
| LV-LIEPAJA | 0.008 | 0.009 | 0.011 | - | **0.000** | **0.000** | **0.000** | **0.000** | **0.000** | **0.000** | **0.000** | **0.000** | **0.000** | **0.000** | **0.000** |
| EE-MUDASTE | 0.008 | 0.007 | 0.013 | 0.005 | - | 0.411 | 0.414 | 0.295 | **0.000** | 0.484 | **0.012** | **0.000** | 0.039 | **0.031** | **0.033** |
| SE-BLEKINGE | 0.009 | 0.009 | 0.011 | 0.005 | 0.000 | - | 0.561 | 0.505 | **0.000** | 0.277 | **0.011** | **0.000** | 0.290 | 0.144 | **0.014** |
| SE-KALMARSUND | 0.007 | 0.008 | 0.010 | 0.006 | 0.001 | -0.001 | - | 0.151 | **0.000** | 0.191 | **0.002** | **0.000** | 0.249 | **0.018** | 0.061 |
| FI-BROMARV | 0.007 | 0.008 | 0.012 | 0.006 | 0.003 | 0.000 | 0.001 | - | **0.000** | 0.866 | 0.084 | **0.005** | 0.734 | 0.202 | 0.158 |
| EE-NARVANLAHTI | 0.016 | 0.018 | 0.022 | 0.012 | 0.008 | 0.006 | 0.008 | 0.006 | - | **0.000** | **0.000** | **0.000** | **0.000** | **0.000** | **0.000** |
| FI-VIROJOKI | 0.007 | 0.006 | 0.013 | 0.004 | 0.001 | 0.002 | -0.001 | 0.000 | 0.010 | - | 0.118 | **0.000** | 0.768 | **0.024** | 0.377 |
| FI-ECKERO | 0.011 | 0.010 | 0.016 | 0.007 | 0.003 | 0.002 | 0.003 | 0.003 | 0.010 | 0.002 | - | **0.000** | **0.015** | **0.000** | 0.051 |
| FI-VAASA | 0.021 | 0.023 | 0.024 | 0.016 | 0.010 | 0.008 | 0.009 | 0.007 | 0.009 | 0.011 | 0.009 | - | **0.004** | **0.000** | **0.000** |
| SE-UMEA | 0.007 | 0.007 | 0.013 | 0.004 | 0.002 | 0.001 | 0.000 | -0.000 | 0.007 | -0.001 | 0.002 | 0.007 | - | 0.094 | 0.283 |
| FI-SIMO | 0.007 | 0.010 | 0.014 | 0.007 | 0.004 | 0.002 | 0.002 | 0.003 | 0.010 | 0.004 | 0.006 | 0.010 | 0.001 | - | **0.015** |
| SE-LULEA | 0.004 | 0.007 | 0.013 | 0.006 | 0.003 | 0.002 | 0.001 | 0.004 | 0.010 | 0.000 | 0.002 | 0.013 | 0.001 | 0.003 | - |

1. Locus Her14

|  | SE-STROMSTAD | DK-FREDRIKSH. | DE-RUGEN | LV-LIEPAJA | EE-MUDASTE | SE-BLEKINGE | SE-KALMARSUND | FI-BROMARV | EE-NARVANLAHTI | FI-VIROJOKI | FI-ECKERO | FI-VAASA | SE-UMEA | FI-SIMO | SE-LULEA |
| --- | --- | --- | --- | --- | --- | --- | --- | --- | --- | --- | --- | --- | --- | --- | --- |
| SE-STROMSTAD | - | 0.696 | **0.000** | 0.228 | **0.000** | **0.001** | **0.000** | **0.000** | **0.000** | **0.000** | **0.000** | **0.000** | **0.000** | **0.000** | **0.000** |
| DK-FREDRIKSHAVN | 0.000 | - | **0.000** | 0.153 | **0.000** | **0.000** | **0.000** | **0.000** | **0.000** | **0.000** | **0.000** | **0.000** | **0.000** | **0.000** | **0.000** |
| DE-RUGEN | 0.148 | 0.099 | - | **0.000** | **0.000** | **0.000** | **0.000** | **0.000** | **0.000** | **0.000** | **0.000** | **0.000** | **0.000** | **0.000** | **0.000** |
| LV-LIEPAJA | 0.013 | 0.021 | 0.222 | - | 0.038 | 0.032 | 0.030 | **0.004** | 0.055 | **0.013** | **0.000** | **0.000** | **0.009** | **0.003** | **0.002** |
| EE-MUDASTE | 0.094 | 0.104 | 0.353 | 0.022 | - | 0.656 | 0.886 | 0.458 | 0.500 | 0.849 | 0.355 | **0.008** | 0.878 | 0.368 | 0.039 |
| SE-BLEKINGE | 0.055 | 0.078 | 0.321 | 0.006 | -0.002 | - | 0.816 | 0.878 | 0.559 | 0.794 | 0.596 | 0.034 | 0.851 | 0.637 | 0.163 |
| SE-KALMARSUND | 0.088 | 0.106 | 0.361 | 0.020 | -0.010 | -0.006 | - | 0.795 | 0.917 | 0.973 | 0.617 | 0.051 | 0.873 | 0.715 | 0.201 |
| FI-BROMARV | 0.067 | 0.093 | 0.344 | 0.013 | -0.002 | -0.012 | -0.008 | - | 0.593 | 0.922 | 0.927 | 0.106 | 0.773 | 0.987 | 0.387 |
| EE-NARVANLAHTI | 0.082 | 0.104 | 0.360 | 0.018 | -0.005 | -0.007 | -0.010 | -0.009 | - | 0.885 | 0.320 | 0.163 | 0.415 | 0.538 | 0.339 |
| FI-VIROJOKI | 0.080 | 0.102 | 0.356 | 0.018 | -0.007 | -0.009 | -0.011 | -0.010 | -0.010 | - | 0.776 | 0.128 | 0.793 | 0.952 | 0.376 |
| FI-ECKERO | 0.084 | 0.108 | 0.362 | 0.023 | -0.005 | -0.008 | -0.009 | -0.011 | -0.007 | -0.010 | - | 0.055 | 0.745 | 0.938 | 0.174 |
| FI-VAASA | 0.067 | 0.110 | 0.367 | 0.029 | 0.034 | 0.006 | 0.018 | 0.002 | 0.010 | 0.011 | 0.012 | - | **0.010** | 0.145 | 0.974 |
| SE-UMEA | 0.084 | 0.100 | 0.350 | 0.019 | -0.011 | -0.008 | -0.011 | -0.008 | -0.007 | -0.010 | -0.010 | 0.023 | - | 0.630 | 0.062 |
| FI-SIMO | 0.081 | 0.107 | 0.363 | 0.021 | -0.004 | -0.009 | -0.009 | -0.012 | -0.009 | -0.011 | -0.011 | 0.007 | -0.009 | - | 0.465 |
| SE-LULEA | 0.050 | 0.093 | 0.343 | 0.021 | 0.036 | 0.003 | 0.020 | 0.001 | 0.011 | 0.012 | 0.013 | -0.009 | 0.023 | 0.007 | - |
