# Supplementary material for: Oceanographic connectivity and environmental correlates of genetic structuring in Atlantic herring in the Baltic Sea
Source: Evol Appl. 2013 Feb 4;6(3):549–67. doi: 10.1111/eva.12042 (PMC3673481; doi:10.1111/eva.12042)

**Supporting Information 10: Structure results.** Graphs show Evanno's Delta K (Evanno et al. 2005) and the mean log likelihood of K (number of clusters) for all 60 loci, 59 loci, and locus Her14, for K = 1-10 under no admixture, for the dataset containing all sites, and the dataset excluding the DE-RUGEN, SE-STROMSTAD, DK-FREDRIKSHAVN, LV-LIEPAJA cluster.


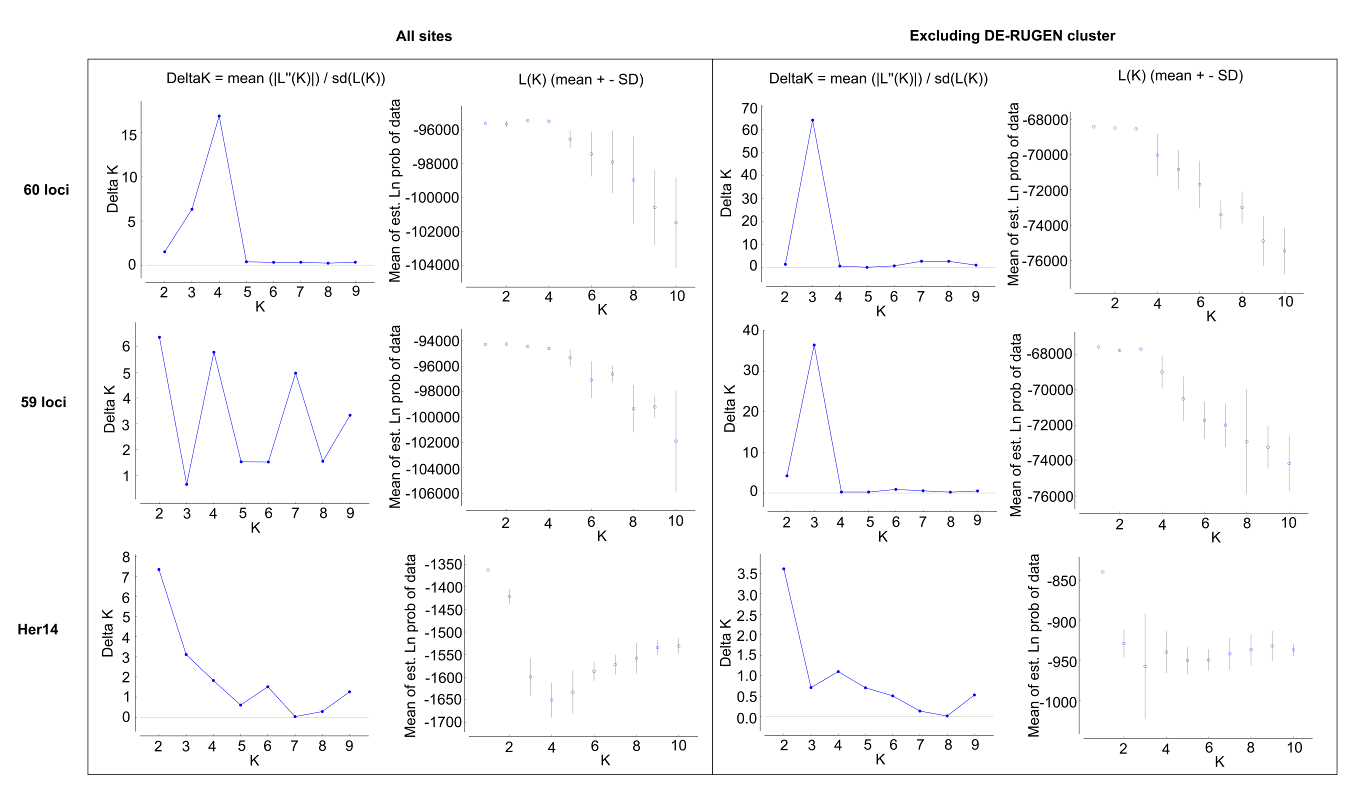

Supplement: Figure S2 — Structure results. [file eva0006-0549-sd10.doc]
