# Supplementary material for: Oceanographic connectivity and environmental correlates of genetic structuring in Atlantic herring in the Baltic Sea
Source: Evol Appl. 2013 Feb 4;6(3):549–67. doi: 10.1111/eva.12042 (PMC3673481; doi:10.1111/eva.12042)
Supplement: Figure S3 — Bayesian clustering results from Structure for outlier loci. [file eva0006-0549-sd11.docx]

**Supporting Information 11: Bayesian clustering results from Structure for outlier loci.** Bar plots are shown for the chosen values of K for the following datasets: (a) positive outlier loci, (b) putative balancing selection outlier loci, (c) positive and putative balancing selection outlier loci, (d) all loci except positive outliers, (e) all loci except putative balancing selection outliers, and (f) all loci except positive and putative balancing selection outliers. Plots are shown including all sites (plots on the left-hand side) and for runs where the DE-RUGEN, SE-STROMSTAD, DK-FREDRIKSHAVN, LV-LIEPAJA cluster was omitted (plots on the right-hand side). Within each plot, each vertical bar represents an individual, clusters are indicated by colour, and the y-axis of each plot shows the proportion of the genotype for each individual belonging to each cluster.**
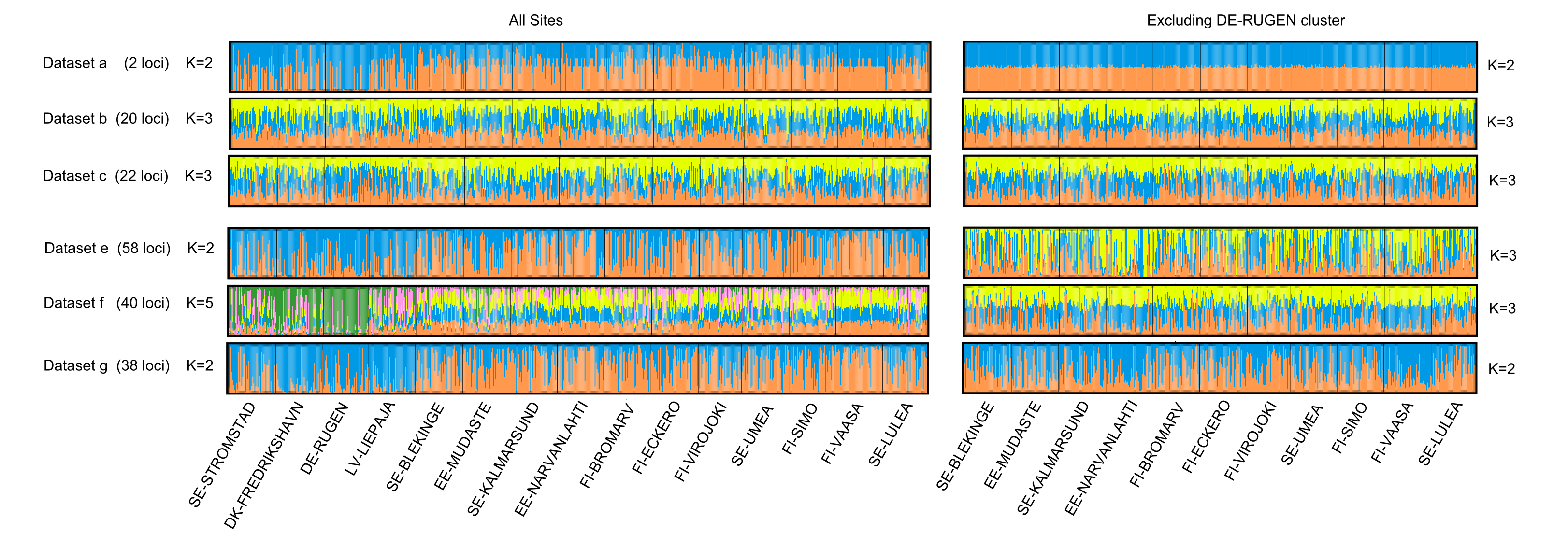
**
