# Supplementary material for: Oceanographic connectivity and environmental correlates of genetic structuring in Atlantic herring in the Baltic Sea
Source: Evol Appl. 2013 Feb 4;6(3):549–67. doi: 10.1111/eva.12042 (PMC3673481; doi:10.1111/eva.12042)
Supplement: Figure S4 — Isolation by distance plots. [file eva0006-0549-sd12.doc]

**Supporting Information 12: Isolation by distance plots.** Plots are shown for the 60 loci, 59 loci, and Her14 locus datasets, including all sites and excluding DE-RUGEN.

**
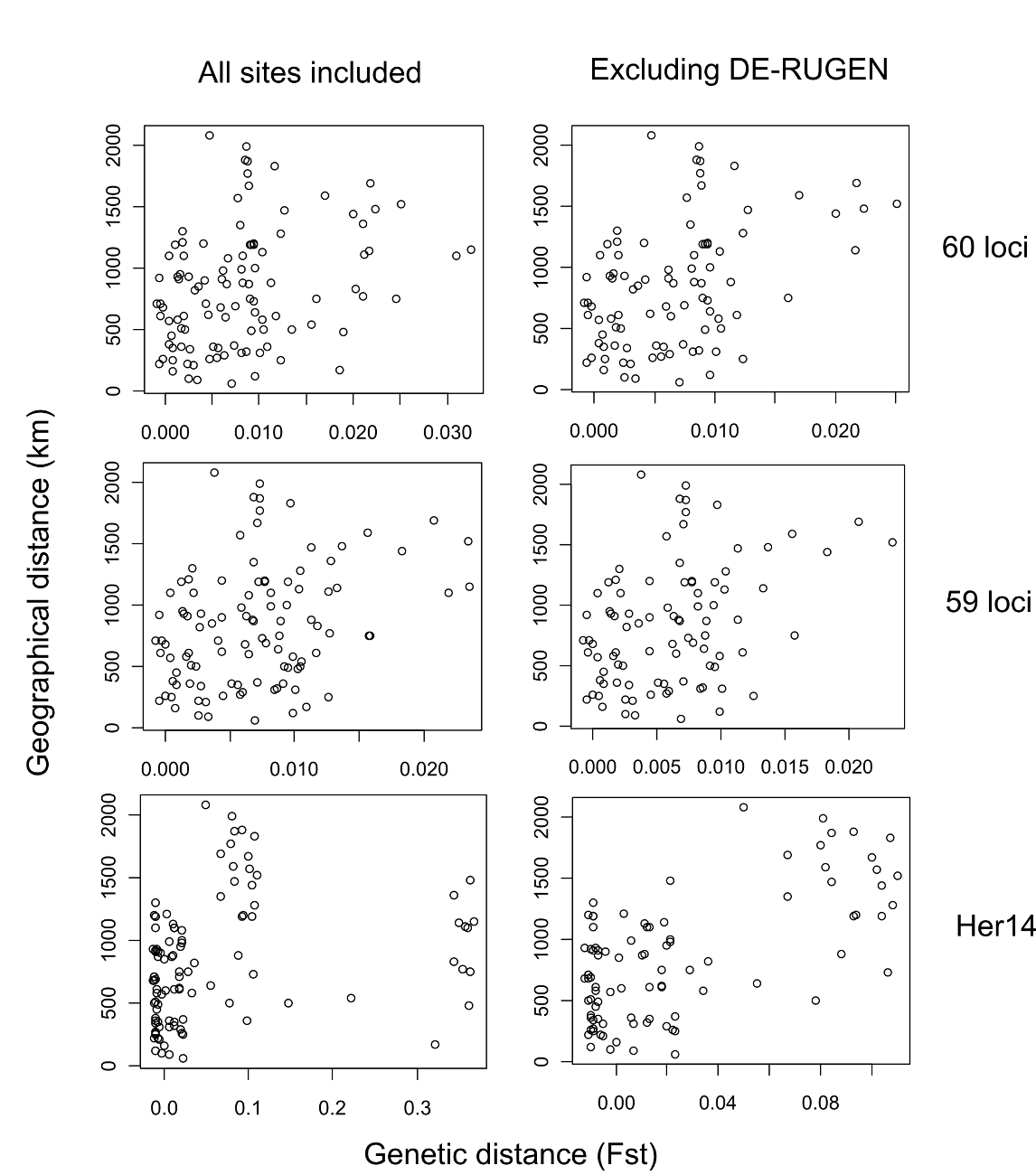
**
