# Supplementary material for: Oceanographic connectivity and environmental correlates of genetic structuring in Atlantic herring in the Baltic Sea
Source: Evol Appl. 2013 Feb 4;6(3):549–67. doi: 10.1111/eva.12042 (PMC3673481; doi:10.1111/eva.12042)
Supplement: Figure S5 — Plots of environmental data. [file eva0006-0549-sd13.docx]

**Supporting Information 13: Plots of environmental data.** These plots show the mean April temperature (ºC) and salinity (‰), and the mean spawning-time temperature and salinity for each of the sites. **
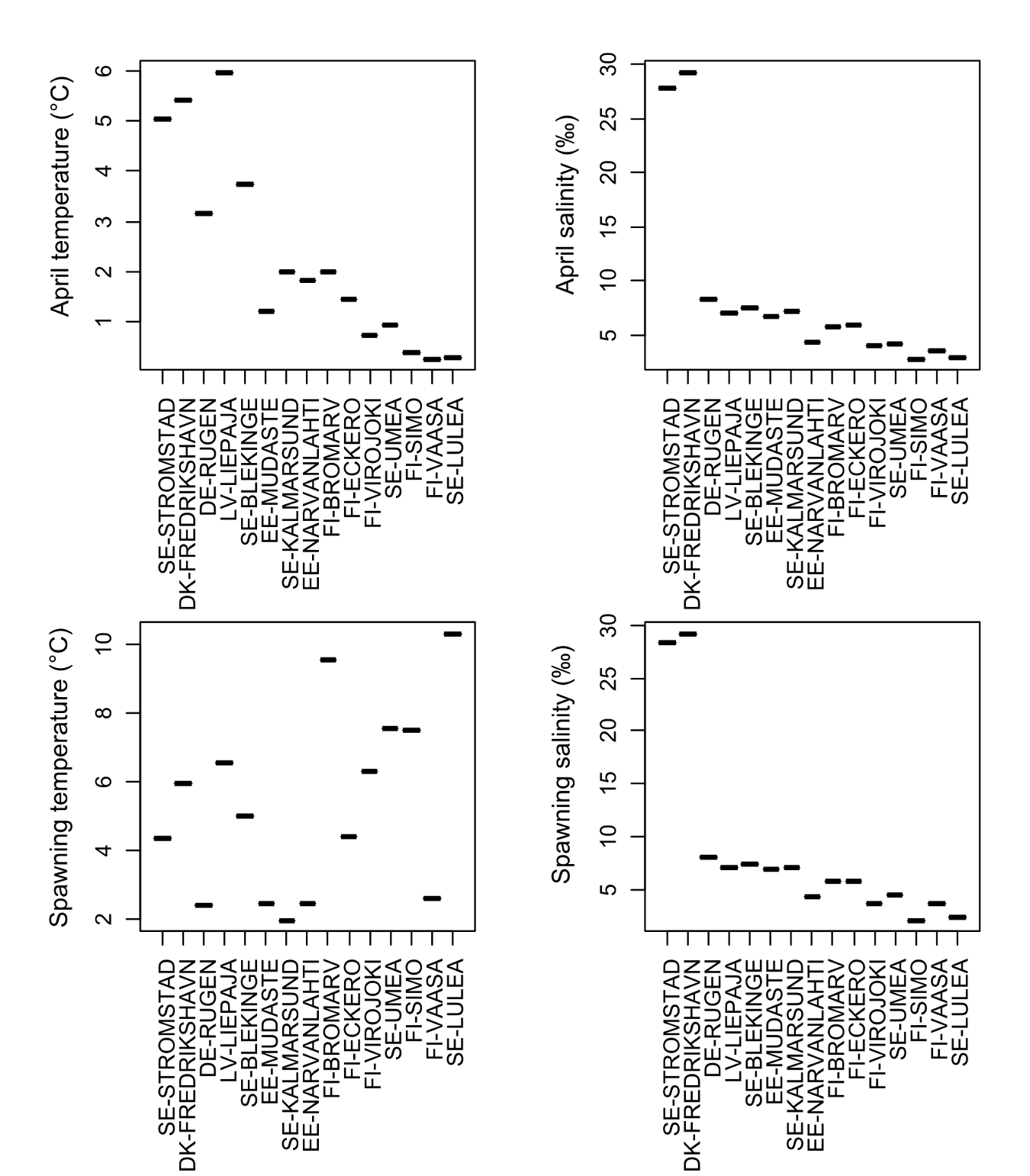
**
