# Supplementary material for: Oceanographic connectivity and environmental correlates of genetic structuring in Atlantic herring in the Baltic Sea
Source: Evol Appl. 2013 Feb 4;6(3):549–67. doi: 10.1111/eva.12042 (PMC3673481; doi:10.1111/eva.12042)
Supplement: Table S9 — Results from general linear models. [file eva0006-0549-sd14.doc]

**Supporting Information 14: Results from general linear models.** With either Allelic Richness (*A*_R_) or expected heterozygosity (*H*_E_) set as the response variables, using datasets consisting of all sites, or all sites excluding DE-RUGEN (excl. DE-RUGEN). For each explanatory variable, the *F* statistic (*F*_1,6_) and associated probability value (*p*) are given. Probability values *p*<0.05 are shown in bold, however these become non-significant at a False Discovery Rate of 0.05, with adjusted *p*-values of (a) 0.082 and (b) 0.071.

|  | | | Fishing pressure | | Distance from entrance of Baltic | | April temp | | April salinity | | Spawn temp | | Spawn salinity | | April temp:April salinity | | Spawn temp:Spawn salinity | |
| --- | --- | --- | --- | --- | --- | --- | --- | --- | --- | --- | --- | --- | --- | --- | --- | --- | --- | --- |
|  |  |  | *F* | *p* | *F* | *p* | *F* | *p* | *F* | *p* | *F* | *p* | *F* | *p* | *F* | *p* | *F* | *p* |
| 60 loci | *A*_R_ | All sites | 2.584 | 0.169 | 0.001 | 0.982 | 0.308 | 0.599 | 0.012 | 0.918 | 5.146 | 0.064 | 0.003 | 0.962 | 1.895 | 0.241 | 0.941 | 0.387 |
|  |  | Excl. DE-RUGEN | 2.984 | 0.159 | 0.222 | 0.658 | 0.679 | 0.448 | 0.18 | 0.689 | 5.028 | 0.075 | 0.172 | 0.695 | 0.534 | 0.518 | 1.278 | 0.341 |
|  | *H*_E_ | All sites | 3.486 | 0.121 | 0.002 | 0.968 | 0.024 | 0.883 | 1.859 | 0.222 | 5.497 | 0.057 | 1.689 | 0.241 | 0.266 | 0.633 | 1.348 | 0.31 |
|  |  | Excl. DE-RUGEN | 2.811 | 0.169 | 0.001 | 0.976 | 0.03 | 0.87 | 1.345 | 0.299 | 4.629 | 0.084 | 1.172 | 0.328 | 0.27 | 0.639 | 0.842 | 0.427 |
| 59 loci | *A*_R_ | All sites | 2.558 | 0.171 | 0.001 | 0.978 | 0.31 | 0.598 | 0.016 | 0.904 | 5.27 | 0.061 | 0.006 | 0.942 | 1.96 | 0.234 | 0.954 | 0.384 |
|  |  | Excl. DE-RUGEN | 2.93 | 0.162 | 0.266 | 0.628 | 0.575 | 0.449 | 0.192 | 0.68 | 5.134 | 0.073 | 0.189 | 0.682 | 0.57 | 0.505 | 1.273 | 0.341 |
|  | *H*_E_ | All sites | 4.155 | 0.097 | 0.008 | 0.931 | 0.001 | 0.973 | 1.735 | 0.236 | 5.231 | 0.062 | 1.592 | 0.254 | 0.345 | 0.589 | 1.467 | 0.292 |
|  |  | Excl. DE-RUGEN | 3.352 | 0.141 | 0.001 | 0.983 | 0.004 | 0.955 | 1.27 | 0.311 | 4.387 | 0.09 | 1.121 | 0.338 | 0.326 | 0.608 | 0.924 | 0.407 |
| Her14 | *A*_R_ | All sites | 2.29 | 0.191 | 2.762 | 0.148 | 0.232 | 0.648 | 0.221 | 0.655 | 1.233 | 0.309 | 0.482 | 0.513 | 0.46 | 0.535 | 0.474 | 0.529 |
|  |  | Excl. DE-RUGEN | 2.809 | 0.169 | 1.072 | 0.348 | 0.713 | 0.437 | 0.01 | 0.925 | 1.199 | 0.323 | 0.072 | 0.799 | 0 | 1 | 1.099 | 0.372 |
|  | *H*_E_ | All sites | 3.309 | 0.129 | 1.286 | 0.3 | 14.518 | **0.009a** | 0.417 | 0.542 | 1.675 | 0.243 | 0.296 | 0.606 | 5.217 | 0.084 | 0.008 | 0.934 |
|  |  | Excl. DE-RUGEN | 3.841 | 0.122 | 0.294 | 0.611 | 17.872 | **0.008b** | 0.094 | 0.771 | 2.159 | 0.202 | 0.02 | 0.892 | 2.381 | 0.221 | 0.003 | 0.962 |
