# Supplementary material for: Oceanographic connectivity and environmental correlates of genetic structuring in Atlantic herring in the Baltic Sea
Source: Evol Appl. 2013 Feb 4;6(3):549–67. doi: 10.1111/eva.12042 (PMC3673481; doi:10.1111/eva.12042)
Supplement: Table S10 — Comparison between present study and previous studies, using the same nine microsatellites. [file eva0006-0549-sd15.doc]

**Supporting Information 15: Comparison between present study and previous studies, using the same nine microsatellites.** (1) present study, (2) Bekkevold et al 2005, (3) Jørgensen et al 2005 (uses Cpa107 instead of Cpa113).

| Comparison using 9 loci | *F*_ST_ |
| --- | --- |
| DE-RUGEN 2002/3 vs SE-STROMSTAD 2002/3 (2) | 0.010 |
| DE-RUGEN 2010 vs SE-STROMSTAD 2009 (1) | 0.009 |
| DE-RUGEN 2002 vs FI-BROMARV 2002 (3) | 0.018 |
| DE-RUGEN 2003 vs FI-BROMARV 2003 (3) | 0.012 |
| DE-RUGEN 2010 vs FI-BROMARV 2009 (1) | 0.007 |
| DE-RUGEN 2002 vs FI-SIMO 2002 (3) | 0.011 |
| DE-RUGEN 2010 vs FI-SIMO 2010 (1) | 0.013 |
